# pH-Sensitive In Situ Gel of Mirtazapine Invasomes for Rectal Drug Delivery: Protruded Bioavailability and Anti-Depressant Efficacy

**DOI:** 10.3390/ph17080978

**Published:** 2024-07-24

**Authors:** Essam M. Eissa, Amani M. El Sisi, Marina A. Bekhet, Fatma I. Abo El-Ela, Rasha M. Kharshoum, Adel A. Ali, Majed Alrobaian, Ahmed M. Abdelhaleem Ali

**Affiliations:** 1 Department of Pharmaceutics and Industrial Pharmacy, Faculty of Pharmacy, Beni-Suef University, Beni-Suef 62514, Egypt; essam.mohamed@pharm.bsu.edu.eg (E.M.E.); amani_mcc@yahoo.com (A.M.E.S.); marinaadelnoshey@pharm.bsu.edu.eg (M.A.B.); rasha0mohd@hotmail.com (R.M.K.); adel.ali@pharm.bsu.edu.eg (A.A.A.); 2 Department of Pharmacology, Faculty of Veterinary Medicine, Beni-Suef University, Beni-Suef 62511, Egypt; fa.pharma@yahoo.com; 3 Department of Pharmaceutics and Industrial Pharmacy, College of Pharmacy, Taif University, Taif 21944, Saudi Arabia; majed.alrobaian@tu.edu.sa

**Keywords:** depression, invasomes, pharmacokinetics, pharmacodynamics, rectal delivery

## Abstract

The present research emphasizes fabrication alongside the assessment of an innovative nano-vesicular membranous system known as invasomes (NVMs) laden with Mirtazapine for rectal administration. This system could circumvent the confines of orally administered counterparts regarding dose schedules and bioavailability. Mirtazapine invasomes were tailored by amalgamating phospholipid, cineole, and ethanol through a thin-film hydration approach rooted in the Box–Behnken layout. Optimization of composition parameters used to fabricate desired NVMs’ physicochemical attributes was undertaken using the Design-Expert^®^ program. The optimal MRZ-NVMs were subsequently transformed to a pH-triggered in situ rectal gel followed by animal pharmacodynamic and pharmacokinetic investigations relative to rectal plain gel and oral suspension. The optimized NVMs revealed a diameter size of 201.3 nm, a z potential of −28.8 mV, an entrapment efficiency of 81.45%, a cumulative release within 12 h of 67.29%, and a cumulative daily permeated quantity of 468.68 µg/cm^2^. Compared to the oral suspension, pharmacokinetic studies revealed a 2.85- and 4.45-fold increase in calculated rectal bioavailability in circulation and brain, respectively. Pharmacodynamic and immunohistopathology evaluations exposed superior MRZ-NVMs attributed to the orally administered drug. Consequently, rectal MRZ-NVMs can potentially be regarded as a prospective nanoplatform with valuable pharmacokinetics and tolerability assets.

## 1. Introduction

Depression is a widespread physiological problem that undermines human emotion, cognition, and behavior. Depression is estimated to affect 3.8% of the population, with adults constituting 5% and those aged 60 and above comprising 5.7% [1,2]. While one of the most ordinarily agreed-upon theories is disparate brain chemistry, depression is caused by an intricate merger of psychological, social, and biological factors. People experiencing depression appear to have a skewed mindset, with a strong sense of grief, anxiousness, suicidal desires, disrupted sleep, and a loss of interest in formerly enjoyable activities throughout the day [3].

Mirtazapine (MRZ) is an unorthodox tetracyclic antidepressant primarily assigned to treat moderate to serious depression and restlessness. MRZ was synthesized by experts in 1989 and approved for severe depression in the Netherlands in 1994. The US FDA approved it for severe mood disorder therapy in 1996 [4,5]. MRZ is an antidepressant with an innovative mechanism: (1) it inhibits central α2-adrenergic auto- and heteroreceptors, thus boosting noradrenergic and serotonergic neurotransmission. (2) It induces the unleash of serotonin (5-hydroxytryptamine; 5-HT) through the stimulation of serotonin 5-HT1 receptors and direct obstruction of 5-HT2 and 5-HT3 receptors. (3) It inhibits histamine H1 receptors but has little anticholinergic action [6]. Along these lines, MRZ’s antidepressant dual action is assumed to be due to an increase in noradrenergic and 5-HT1 receptor-mediated neurotransmission [7,8]. The drug itself is tranquilizing, antiemetic, anxiolytic, and an appetite promoter. MRZ is frequently administered to melancholy patients with trouble sleeping and underweight ones [5].

Thus far, MRZ is currently only accessible in the market in oral tablet form. This oral dosage form exhibits an array of drawbacks in terms of both dosing regimen and pharmacokinetic characteristics. The primary encumbrance is that its absolute bioavailability is only around 50%, owing mostly to the gut wall and liver first-pass metabolic process. Its metabolism is mostly mediated by the cytochrome P450 (CYP) isoenzymes CYP2D6, CYP1A2, and CYP3A4 [9]. Secondly, oral administration also presents challenges due to food in the stomach, which may impede medication absorption. This is especially true for fatty meals since MRZ is a highly lipophilic drug with a partition coefficient of 2.9 [6]. Long-term MRZ usage causes many side effects. Weight gain and sedation are noticeable. MRZ has also been linked to hematologic and cardiovascular adverse effects [10]. Not to mention, an alternative delivery route might be regarded as a critical requirement to blow away the concomitant oral hindrance.

Rectal medication administration is often regarded as a noninvasive, tailored approach that has the potential to alleviate systemic toxicity. It is also being appraised for systemic drug delivery, thanks to recently developed drug delivery strategies. The rectal avenue possesses various benefits, including lower susceptibility to enzymatic breakdown and the possibility of partly mitigating the medications’ presystemic digestion. As a con-sequence, medication absorption was stabilized, ensuring stable plasma drug levels, minimizing dosage intervals, and enhancing patient satisfaction [11,12]. Conventional suppositories may prompt patient discomfort and repudiation, leading to reduced patient compliance. Furthermore, if they lack mucous membrane adhesion, such traditional doses can readily reach the distal end of the colon, subjecting the loaded medications to first-pass digestion. As it stands, an alternative simple-to-administer rectal preparation of sufficient bioadhesive strength has triggered curiosity [13,14].

A pH-responsive in situ hydrogel system based on Chitosan (CH) and Glyceryl monooleate (GMO) has many benefits, including boosted mucoadhesion [15]. CH is a naturally occurring mucoadhesive polymer that can enhance targeted and extended medication administration. The relevance of its incorporation is that acidic CH solutions produce viscous gels when exposed to alkaline pH. Also, CH’s positive charges may cause a robust electrostatic attraction with mucosal surface that is negatively charged [16]. Meanwhile, GMO develops a clear, rigid, gelatinous cubic state with a three-dimensional framework of bent lipid bilayers in the presence of sufficient water [17]. Collectively, the synergistic impact of the CH/GMO pH-triggered in situ gelling system is beneficial for medication targeting, ensuring the easy application of the rectal dosage and maintenance [18].

Phospholipid nano-systems have sparked huge interest, notably as a promising drug delivery technique capable of enhancing the absorption of many drugs while avoiding loss in the GI tract and liver [19,20]. Liposomes, the initial iteration of phospholipid-based vesicles, could be considered pioneers in this field. Constructed from one or more lipid bilayers encircling an aqueous core, they have the capacity to encapsulate a vast array of hydrophilic or lipophilic pharmacological compounds [21]. Unexpectedly, numerous additives, such as alcohol and surfactants, have been employed to surrogate the chemically based, tangible, and functioning features of liposomes over the past decades, and more advanced iterations (known to be malleable or highly elastic liposomes) were promoted, overcoming biological limitations to achieve improved medication delivery. Glycerosomes, transferosomes, ethosomes, and invasomes are illustrations of these novel mechanisms that have been intensively investigated for the transmucosal and rectal delivery of an assortment of drugs [20,22,23,24,25,26].

Invasomes (NVMs) are novel elastic phospholipid paradigms built of phosphatidylcholine, ethanol, and one or consolidation of terpenes. Many studies have already demonstrated terpenes’ capacity to revamp membrane penetration [27,28]. By disrupting surface lipids, interacting with intracellular proteins, and reversing drug partitioning across mucosal membranes, they are capable of enhancing penetration. In addition, ethanol’s net negative surface charge prevents electrostatic repulsion-induced vesicle aggregation [29,30]. Hence, terpenes and ethanol proved to have a harmonious impact on drug permeation [31,32].

Overall, the current study endeavored to investigate the prospect customization of a novel NVM strategy for the effective delivery of MRZ to target the brain via a more expedient pathway, the rectal route, in order to increase bioavailability and sustain the therapeutic effect while avoiding the superfluous limitations linked to the oral route. In this prospect, NVM formulations were tailored and physicochemically evaluated using the Box–Behnken design. Based on the optimal formulation, a pH-triggered in situ gel-forming system was built. Rectal tolerance and toxicology experiments on rats were carried out to rule out any potentially harmful effects on the rectal mucosa. Finally, the optimal rectal MRZ-NVM in situ gelling system’s in vivo pharmacodynamics and pharmacokinetic behavior in rats were determined compared to the corresponding rectal-free MRZ in situ gel and oral drug suspension.

## 2. Results

### 2.1. MRZ Entrapment Efficiency and Vesicle Size Analysis

**Figure 1:**
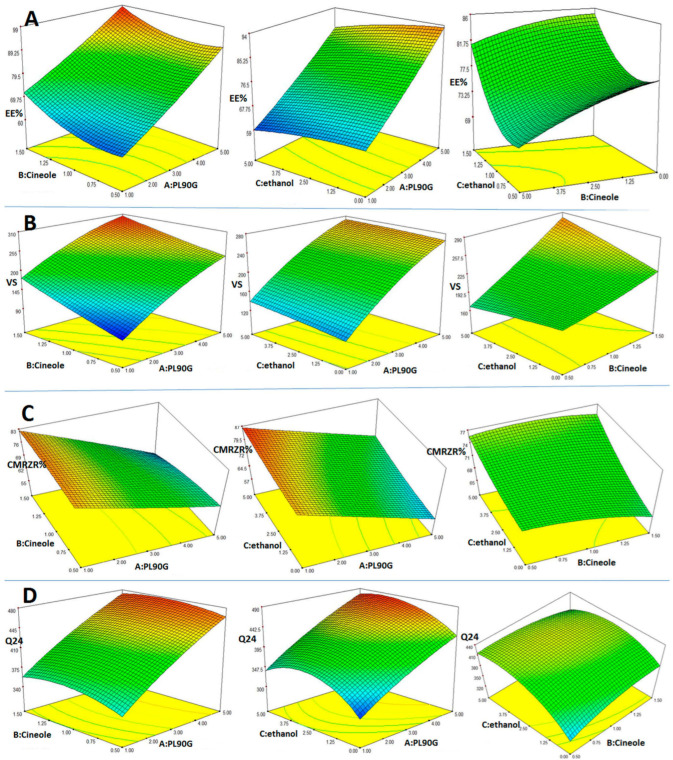
The 3D surface response diagrams showing the contribution of the three variables on (**A**) EE%, (**B**) vesicle size, (**C**) CMRZR%, and (**D**) Q24.

As apparent from Table 1, the nano-invasomal cargo picked up MRZ, with an EE% oscillating between 59.41 ± 2.18 and 97.15 ± 1.40%. The quadratic model proved to be the ideal fit for assembling the EE% data (highest R^2^) (Supplementary Table S1). The results were significantly different (*p* < 0.0001), with a high F-value of 107.61 noted. Figure 1A shows the combined impact of two causative factors on the EE% of MRZ (Y1) in the customized NVMs at the middle level of the third variable as 3D surface plots. ANOVA study of the experimental findings reveals a regression equation: Equation (1), which interrelates the ramifications of the three causative variables on the EE% of MRZ in terms of coded values.

EE% = +75.47 + 14.19A + 4.69B − 2.90C + 3.95B^2^ (1)

MRZ-NVMs formulation VS values equivocated from 88.97 ± 7.9 to 307.83 ± 6.2 nm, as observed in Table 1. PDI values of MRZ-NVMs fluctuate ≤ 0.434 (Table 2).

Figure 1B depicts the outcome of formulation factors on the VS of MRZ-NVMs. The influence of independent factors on NVMs VS was chosen by adopting a quadratic model with the greatest R^2^ value. The F-value for the invasomal size model was 407.95 (*p* < 0.0001). The numerical contribution of the three variables to the VS of NVMs with encrypted values was represented in Equation (2):

VS = +215.98 + 69.25A + 37.59B + 5.88C − 6.40AB + 18.83BC − 14.35A^2^ (2)

### 2.2. In Vitro Release and Ex Vivo Skin Permeation Studies

The quadratic interaction model exhibited statistical significance (*p* < 0.0001) in relation to the observed CMRZR% data. The model’s F value was 84.18. The three-dimensional surface plot in Figure 1C clearly illustrates the %CMRZR. The quadratic model equation (Equation (3)) of the % CMRZR data with relation to the encoded variables was represented by means of the subsequent equation:

(3) $${\%CMRZR}^{2.07}=+6909.07-2173.3A-278.66B+711.85C-397.97AB$$

All 15 release patterns of the MRZ-NVMs formulations are represented (Supplementary Figure S1) and compared with the release of free MRZ suspension. Kinetic analysis for the formulation release patterns can be found in supplementary data (Supplementary Table S2).

Table 2 presents findings of Q24 varying from 295.68 ± 14.74 to 477.33 ± 15.31 µg/cm^2^ compared to the free MRZ suspension with an equivalent MRZ (Q24 247.06 ± 11.81 µg/cm^2^) (Supplementary Figure S2). The penetration assets of the MRZ-NVMs through the rectal mucosa are shown collectively in Table 2. The quadratic equation exhibited has an F-value of 110.19 and a *p*-value < 0.0001). Equation (4) represents the impact of independent factors on Q24 in encoded values:

(4) $${Q24}^{2.75}=+1.729E+007+6.056E+006A+5.054E+005B+2.234E+006C+1.003E+006AC-1.631E+\;006BC-1.162E+006B^{2}-3.382E+006C^{2}$$

### 2.3. Expert Design Optimization

Sufficient precision was used to calculate the signal-to-noise ratio and ensure the model’s capacity to traverse the design space. All four responses exhibited a ratio greater than four, considered a desirable number, as indicated in Supplementary Table S1. The ANOVA analysis revealed no obvious lack of consistency in any response. The Design Expert^®^ program suggested an optimal formula with the composition of 4.81% *w*/*v* PL90G, 0.5% *v*/*v* cineole, and 5% *v*/*v* ethanol. Based on the formulation, this mixture was selected due to its high desire index (0.553). According to the data shown in Supplementary Table S3, the observed responses derived from the newly developed formulation exhibited a high degree of concordance with the anticipated values, demonstrating an acceptable level of percentage error. Moreover, when considering a significance level of *p* ≤ 0.05, there was no significant difference between the actual and projected values.

### 2.4. Characterization of the Optimized MRZ-NVMs Formulation

**Figure 2:**
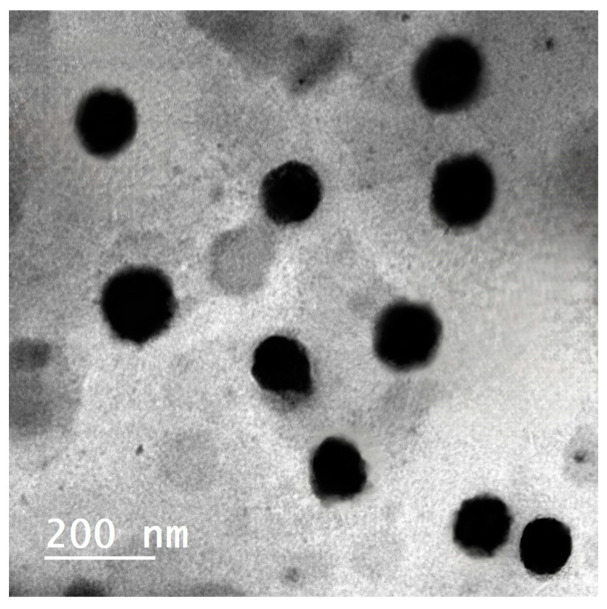
TEM photomicrograph for the optimized MRZ nano-invasomal formulation.

**Figure 3:**
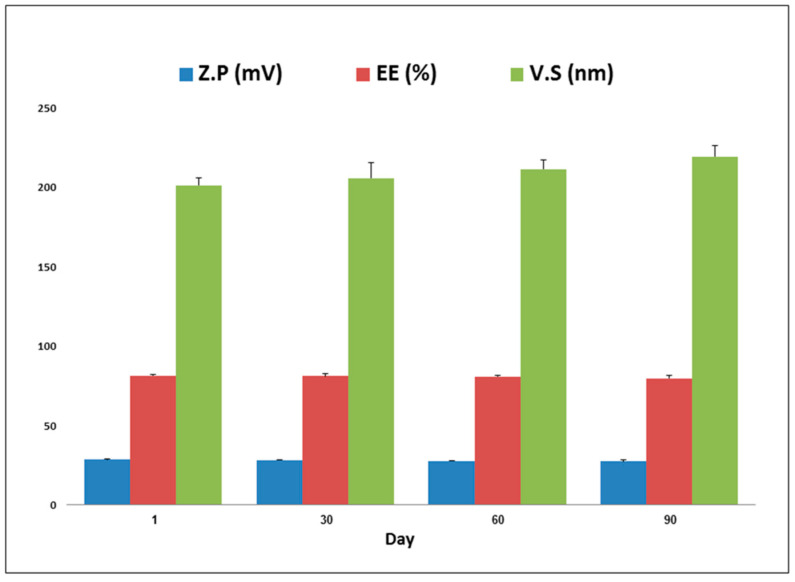
Stability studies of the optimized MRZ nano-invasomal formulation.

The optimized vesicles showed stable, spherical, and approximately monosized vesicles (Figure 2) with an average ZP value of −28.8 ± 0.22 mV (Supplementary Figure S3). The stability analysis demonstrated that there was no significant difference (*p* < 0.05) between the optimized MRZ-NVMs formulation after a storage period of 90 days and the fresh one in values of EE%, invasomal VS, or ZP (Figure 3). The boosted formulation exhibited no precipitation or layer differentiation indications, suggesting acceptable kinetic stability.

Concerning DSC, the crystalline structure of MRZ is demonstrated by a conspicuous abrupt endothermic peak at 115.84 °C appearing in the DSC spectrum (Supplementary Figure S4A). Sharp endothermic peaks were visible in the thermograms of cholesterol and PL90G at 148 °C and 136.4 °C, respectively. These peaks corresponded to their melting temperatures. The appearance of all the indicated peaks was clearly visible on the MRZ–cholesterol, MRZ-PL90G, and MRZ–cineole combination thermograms, with no significant alterations.

Supplementary Figure S4B displays the FT-IR spectra of MRZ, cholesterol, PL90G, cineole, and ethanol, as well as physical mixtures of MRZ with each of the constituents. MRZ spectra showed bands for C–C stretching of the phenyl group at 1485 cm^−1^ and 1440 cm^−1^; large peaks at 3420 cm^−1^, indicating N–H stretching; and a band at 2900 cm^−1^, resulting from a methyl group attached to N2 atom. The bands at 1370–1280 cm^−1^ were generated by aromatic primary amines where the N atom was attached directly to the ring. The region of 1350–1054 cm^−1^ is where the benzene ring C–H originates. The FT-IR spectra of the MRZ–ethanol, MRZ–cineole, MRZ–cholesterol, and MRZ-PL90G mixtures showed similar peaks.

### 2.5. Characterization and Investigation of the In Situ Gel

The MRZ-NVMs in situ gel exhibited a refined appearance, characterized by its white color and consistent, homogeneous texture, devoid of air bubbles or clumps.

The *sol–gel* transition pH of the optimized MRZ-NVMs in situ gel was determined to be 7.1 ± 0.19, with a gelation duration of 35 s ± 3.2. Additionally, the in situ gel’s MRZ concentration was 98.72 ± 1.35%. The formulation spread readily (5.3 cm in diameter). Furthermore, the bioadhesive force of NVMs in situ gel was determined to be 13,782.5 ± 8.19 dyne/cm^2^. In addition, the tailored MRZ-NVMs in situ gel exhibited low viscosity at acidic pH (1280.03 ± 13.4 cps), and a higher value (7218.08 ± 11.75 cps) at physiological pH equals pH of the rectum (7.4). The gel also had shear thinning capabilities (pseudoplastic), as shown by a Farrows constant ˃1 (N = 2.83 ± 0.14) (Supplementary Figure S5). The hysteresis loop’s area was similarly determined to be 2696.528 and 1889.776 Dyne/cm^2^·s for the in situ gel at pH = 7.4 and pH = 4, respectively.

Regarding the in vitro release analysis and ex vivo diffusion investigations, the findings led to a reduction in the quantity of MRZ released and permeated in comparison to the optimized formulation prior to its integration into the gel (Supplementary Figure S6A,B).

### 2.6. Behavioral Analysis: Immobility, Climbing, Swimming in FST, Open Field Test (OFT), and Tail Suspension Test (TST)

**Figure 4:**
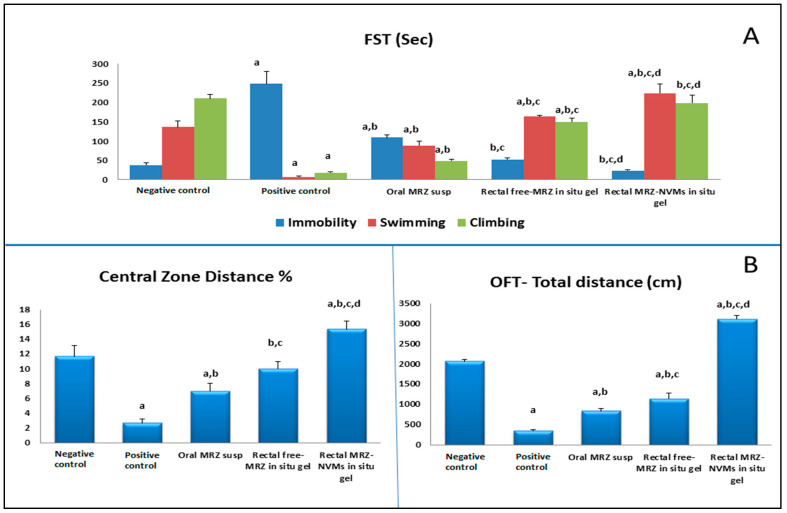
(**A**) FST behavioral analysis (immobility, swimming, and climbing) for MRZ oral suspension and the MRZ-NVM rectal pH-triggered gel preparation in comparison to normal control and positive depressed rats. (**B**) OFT behavioral analysis of the total distance (cm) and the central zone (%) traversed by MRZ oral suspension and the MRZ-NVM rectal pH-triggered gel preparation compared to negative and positive controls. ^a^ *p* < 0.05 relative to the negative control; ^b^ *p* < 0.05 relative to the positive control; ^c^ *p* < 0.05 relative to MRZ-susp (oral); ^d^ *p* < 0.05 relative to free mRZ- in situ rectal gel.

Figure 4A depicts FST results on rats’ escape, leap, climb, and swim abilities. The positive control group was the least mobile, spending a scant amount of time swimming and climbing. Nonetheless, compared to oral MRZ-Susp and other formulations, MRZ-NMVS gel rectally brought about an astounding rise in climbing and swimming durations while concurrently decreasing immobility (*p* < 0.05). Figure 4B shows the data displaying rats’ performance in OFT. The depressed group had markedly lower levels of cross lines and rearing numbers than the normal control group (*p* < 0.05). Meanwhile, compared to the normal control and orally treated groups, the administration of MRZ-NMVs gel resulted in a significant increase in rearing behaviors and % central zone distance.

**Figure 5:**
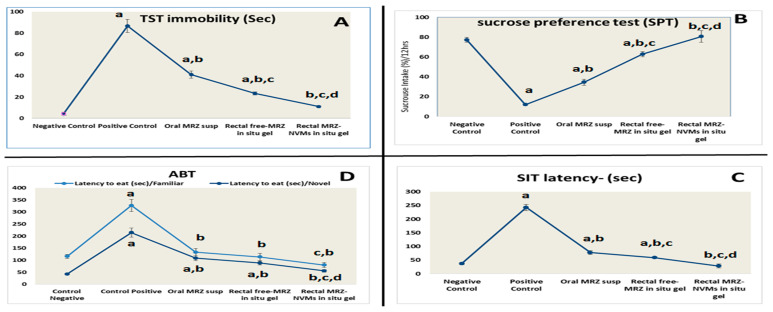
(**A**) Total time in seconds for immobility in TST for oral and rectal preparations compared with normal control and depressed rats. (**B**) Effect of stress of food deprivation in rats (Sucrose Preference Test) within 12 h for oral and rectal preparations and the negative and positive control groups. (**C**) Social interaction latency time in seconds for rats dosed with oral and rectal preparation compared to normal and depressed groups. (**D**) Latency time in seconds for rats to start eating in novel and familiar environments in Anxiety-Based Test (ABT) in oral and rectal groups and compared to negative control and positive groups. ^a^ *p* < 0.05 relative to the negative control; ^b^ *p* < 0.05 relative to the positive control; ^c^ *p* < 0.05 relative to MRZ-susp (oral); ^d^ *p* < 0.05 relative to free mRZ- in situ rectal gel.

The TST findings showed that the nano-formula rectally treated group had a statistically significant reduction in immobility time (*p* < 0.05) (11.00 ± 1.00 s) compared to the positive control group in terms of lowering depressive symptoms (86.6 ± 6.11 s). MRZ-NMVS gel revealed a greater degree of efficiency (*p* < 0.05) than MRZ-Susp (oral) (12.3 ± 2.5 s) (Figure 5A).

### 2.7. Sucrose Preference Test (SPT), Social Interaction Test (SIT), and Anxiety-Based Test (ABT)

Sucrose was consumed by all rats participating in the SPT. On the other hand, the simultaneous administration of multiple MRZ formulations produced various results. As demonstrated in Figure 5B, the MRZ-NMVs gel (80.66 ± 5.85%), MRZ-Susp (34.6 ± 3.05%), and free mRZ-gel (63.2 ± 2.64%) had a statistically significant increase in sucrose intake (*p* < 0.05) when compared to the positive control group (12.00 ± 1%), with leading results for the optimized nano-formulation in G5. In the SIT study outcomes, dejected behavior was assessed using two essential parameters: the extent of interactions and the duration of active interactions. As demonstrated in Figure 5C, the experimental positive group experienced depressive symptoms, which was evidenced by a considerable increase in the latency time to begin socializing (242.3 ± 11.6 s) compared to the control group (37 ± 4.56 s). Rats given MRZ-NMVs gel interacted and engaged more than the control group. Furthermore, these rats showed a shorter interaction onset time, with 28.3 ± 7.6 s latency. MRZ-NMVS gel demonstrated a noteworthy augmentation in enhanced interaction and time required to commence contact relative to free MRZ gel and MRZ-Susp (measured in seconds).

The ANOVA results showed that the MRZ-NMVs gel, MRZ-Susp, and free mRZ-gel groups had significantly lower latency to feed than the depressed group (Figure 5D) (*p* < 0.05). Nevertheless, in relation to the oral MRZ and MRZ-free gel groups, the administration of MRZ-NMVs gel resulted in a significant reduction in the time delay before eating. 

### 2.8. Tolerability and Acute Toxicity Studies

In a rat approach, daily dosing of an amended rectal formulation resulted in insignificant values for toxicity or mortality. There were no statistically significant distinctions between the MRZ-NVMs gel rectally treated group and the monitoring negative group in hematological indicators such as Hb, RBC, MCH, MCV, MCHC, WBCs, lymphocyte, neutrophil, and monocyte, or biochemical parameters such as urea, creatinine, ALT, and AST (Supplementary Figure S7).

### 2.9. Rectal and Brain Histopathological Examination and Immunohistochemistry Analysis

**Figure 6:**
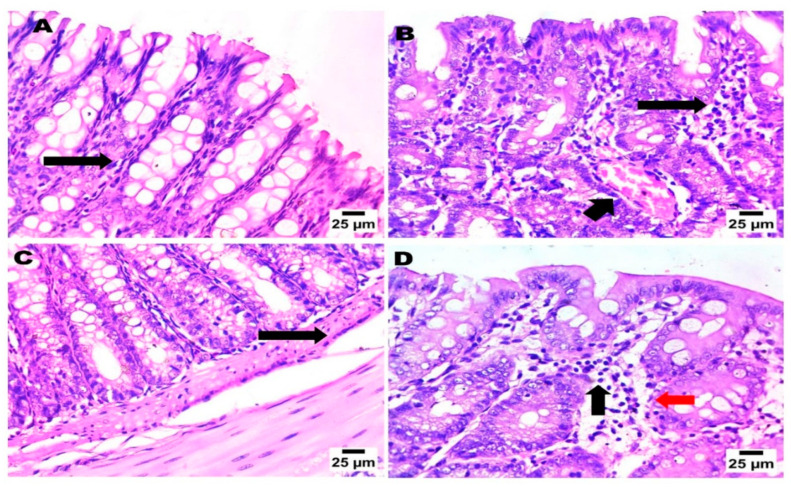
The impact of various treatments, orally administered MRZ (**B**), plain in situ rectal gel (**C**), MRZ-NVM in situ rectal gel (**D**), and compared to negative control (**A**), on the mucosal rectal epithelium histopathology. The rectal epithelium exhibted an intact epithelial lining (black arrow) and a submucosa with average blood vessel denisty with no congestion or inflamation (red arrow).

The study evidenced that there are no harmful effects on the rectal mucosa (Figure 6). Supplementary Figures S8 and S9 represent the cerebral cortex and the hippocampus, respectively, of the three treated groups compared with the negative control group.

**Figure 7:**
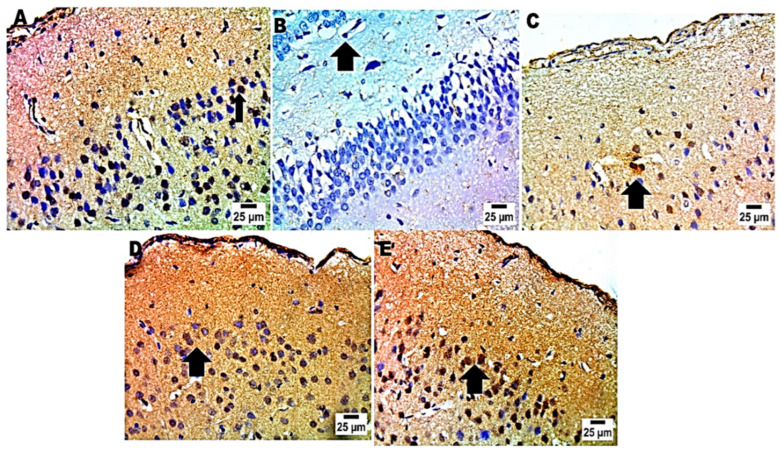
The impact of various interventions: negative control (**A**), positive control (**B**), MRZ–suspension (oral) (**C**), MRZ plain in situ rectal gel (**D**), and MRZ-NVM in situ rectal gel (rectal) (**E**) on the neurological activity and percentage of positive immune cells (black arrows) in the cerebral cortex of the brain.

**Figure 8:**
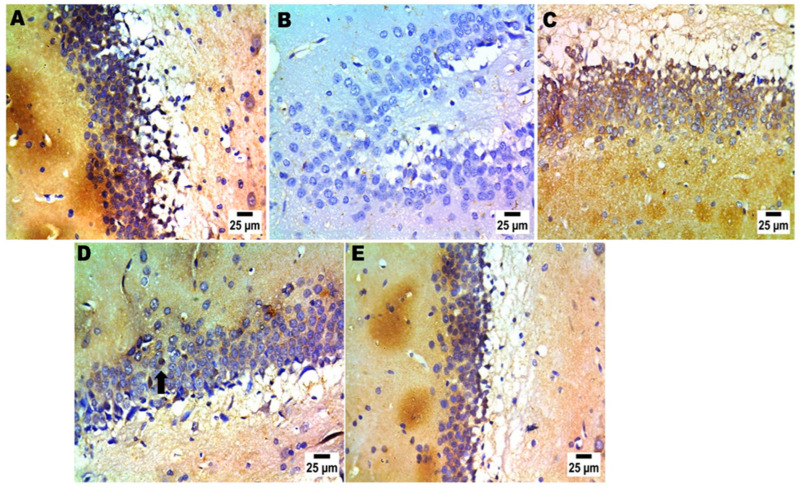
The impact of various interventions: negative control (**A**), positive control (**B**), MRZ–suspension (oral) (**C**), MRZ plain in situ rectal gel (**D**), and MRZ-NVM in situ rectal gel (rectal) (**E**) on the neurological activity and percentage of positive immune cells (black arrows) in the hippocampus of the brain after depression treatment.

In immunohistochemistry analysis, the positive group exhibited the lowest BDNF cell counts in the brain. It is worth noting that the group that provided MRZ-NMVs gel had the greatest density of BDNF cells in cerebral–cortical and hippocampal regions (Figure 7 and Figure 8) and also evidenced a high percentage of scoring (Supplementary Figure S10).

### 2.10. Pharmacokinetics Studies

In order to determine the in vivo pharmacokinetic behavior, the rat plasma MRZ concentrations were quantitatively assessed using the HPLC method, which was validated in accordance with ICH recommendations prior to the pharmacokinetic experiments. The drug’s high linearity was demonstrated by the adopted assay in a matrix-based calibration curve that covered a 200 ng/mL to 1500 ng/mL range (R^2^ = 0.9993). The LLOD was 62 ng/mL, and the lower limit of quantification (LLOQ) in rat plasma was 186 ng/mL. More than 95.46% of the recovery values were achieved with a %R.S.D. of less than 2.16. The accuracy %R.S.D was ≤2.81 and the precision %R.S.D ≤ 3.9. Frequently, these results could demonstrate the linearity, accuracy, reproducibility, and sensitivity of the HPLC method that was devised for the determination of MRZ in rat plasma, as well as its compliance with the pharmacokinetic investigation’s requirements. Supplementary Figure S11 shows distinct chromatograms for the internal standard, MRZ drug, and plasma that clearly show the drug peak and rule out any interference.

**Figure 9:**
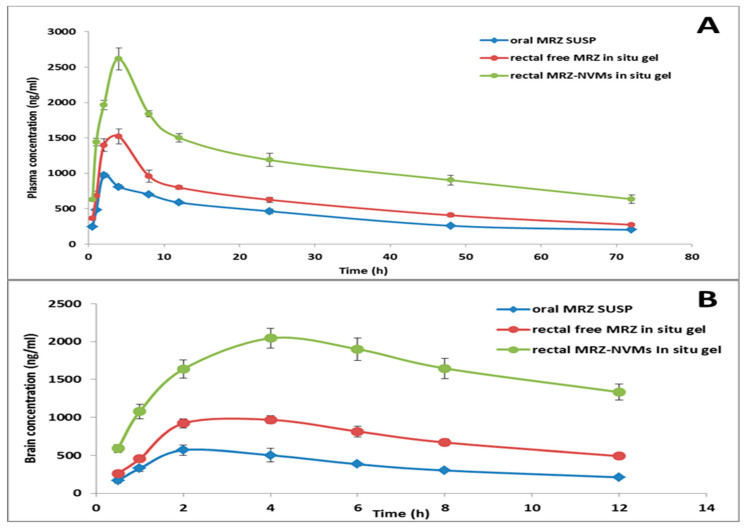
(**A**) Plasma–MRZ level time profiles of MRZ oral suspension, rectal plain in situ gel, and rectal nano-invasomal pH-sensitive gel. (**B**) Brain concentration-time profiles of oral MRZ suspension, rectal plain in situ gel, and rectal nano-invasomal pH-sensitive gel.

MRZ concentrations were assessed at intervals up to 12 and 72 h for the brain and plasma, respectively. Figure 9, in plasma (Figure 9A) and for the brain (Figure 9B), depicts the mean levels of MRZ over time elapsed after rectal and oral delivery, and then many PK variables (Table 3) were computed.

The pharmacokinetic data of the rectally applied optimized MRZ-NVMs in situ gel formulation reveal that the Cmax, t1/2, MRT, and AUC0-∞ were markedly greater (*p* < 0.05) compared to oral MRZ and free MRZ rectally treated groups. Plasma Cmax was found to be 2.69-fold higher after rectal MRZ-NVMs administration (2616.99 ± 127.68 ng/mL) than after oral MRZ administration (972.84 ± 34.28 ng/mL), with AUC0-∞ reaching 130,817.1 ± 7757.3 ng/h/mL and 38,955.64 ± 2051.04 ng/h/mL, respectively. Additionally, rectal administration of MRZ-NVMs gel formulation (brain Cmax = 2064.294 ± 102.94 ng/gm, AUC0-∞ = 43,402.79 ± 6080.81 ng/h/mL) increased MRZ concentration in brain homogenate up to 4.17 times when compared with MRZ suspension. When compared to the plain rectal gel treated group, the nano gel treated group had a 1.28-fold increase in Frel in plasma and a 2.27-fold increase in the brain with a significant increase (*p* < 0.05) in the MRT and AUC0-∞. The percentage compared to the blood and brain bioavailability of MRZ from the rectal MRZ-NVMs in situ gel was approximately 285.40% and 445.1745%, respectively, compared to MRZ’s oral suspension.

It was also found that the optimal MRZ-NVMs in situ rectal gel had a much lower elimination rate (*p* < 0.05) compared to oral MRZ (Table 3). MRZ’s brain/blood ratio was measured at all-time intervals for the rectal and oral formulations (Table 4).

## 3. Discussion

### 3.1. In Vitro Studies and Design Optimization

The design of experiments is an amalgamation of approaches that have been effectively utilized for evaluating the significance of variables and their relations with the empirical results. The response surface approach is a mathematical and statistical tool implemented to reveal a functional articulation between many responses and factors. It could appraise the assessed factors while creating a formula with the sought-after features and effectiveness. In this study, the Box–Benkhen design was adopted since it needed fewer rounds of testing than other strategies, including a full-factorial design [33,34]. The coefficient of determination (R^2^), adjusted (R^2^), predicted (R^2^), and CV% data were employed to determine the best-fit model for each response.

#### 3.1.1. Entrapment Efficiency Optimization

The aptitude of the built innovative NVM system to retain vast quantities of MRZ is critical for its eventual application as a rectal functional delivery approach. In actuality, the physicochemical traits of MRZ imply high lipophilicity; hence, an infallible EE% was deduced. For Equation (1), a positive sign for a regression coefficient signifies that the variables have a synergistic impact, while a negative sign indicates that the factors operate with an antagonistic impact.

Data reveal that PL90G levels have a substantial effect on EE%; increasing the PL90G content in formulations resulted in increased MRZ EE% in nano-invasomes. Furthermore, since the drug remains bound in the lipid phase pursuant to MRZ lipophilicity, raising the proportion of lipid particles forming each vesicle enhances the likelihood of the medication integrating with the invasomes’ lipoidal content [35,36]. Formulations F1, F5, F12, and F15 with the highest percentage of PL90G (5% *w*/*v*) demonstrated greater MRZ EE% than formulations F4, F8, F10, and F13 with the least percentage of PL90G (1% *w*/*v*) (Table 1). With respect to factor B, the noticeable contribution of cineole% to MRZ entrapment efficiency is attributed to cineole’s lipophilicity and molecular structure. It has a high lipophilicity (log *p* = 2.82) [37]. Terpenes in the invasomal lipid bilayer may have a similar impact to PL90G in terms of increasing the area accessible for drug incorporation [38]. Another explanation is that the lipophilic terpene initially dissolves with the PL90G contained within the vesicular bilayer, where the acyl chains of phospholipids provide an appropriate ambiance for the lipophilic terpene and the lipophilic medication. As ensues, the greater the terpene content, the more chains are formed, resulting in enhanced lipophilic drug solubilization in the bilayer and thereby increased EE% [39]. The EE% of formulation F9 (cineole 0.5%) was lower (68.32%) than the EE% of formulation F11 (cineole 1.5%), which was 81.46%. Similar findings were achieved with the formulation F15 (cineole 0.5%), which had a lower EE% (91.27%) than the formulation F5 (cineole 1.5%), which had an EE% of 97.15% (Table 1). Dragicevic-Curic et al. [22] reached the same conclusion in their study of the manufacture of temoporfin-loaded invasomes. Their findings divulge that recruiting more cineole to the prepared invasomes boosts the EE% of temoporfin. The process attribute Y1 (EE%) has an inverse correlation with ethanol. Undoubtedly, with the ethanol concentration abatement, the drug encapsulation grew. This might happen because ethanol provokes seepage, which reduces the nano-invasome’s capacity to retain the medication tightly [40]. Formulation F8 (ethanol 5%) had a lower EE% (59.41%) than Formulation F4 (ethanol 0%), which had a 62.35% encapsulation. The remaining components, such as PL90G and cineole, are in similar proportions in both formulations. The F1 and F12 formulas had matching outcomes.

#### 3.1.2. Vesicle Size Optimization

The VS of nanovesicles affects their penetration through the rectal mucosa; thus, creating small-sized NVMs might considerably boost medication absorption. Adapting small-sized vesicles dampens their surveillance by blood elements, hence extending their circulation duration. Consequently, integrating nano-cargo with tiny vesicle sizes was essential in the present work to ameliorate drug flow and the in vivo biofate of MRZ. Regarding PDI, zero indicates completely monodispersed vesicles, while one indicates an immensely polydispersed vesicle population. PDI values of MRZ-NVMs fluctuate ≤ 0.434, exhibiting restrictive size distributions as well as striking uniformity.

The notion is that the positive sign of the linear term A coefficient implies the PL90G% positive unfavorable role in the response Y2. The 3D graph in Figure 1 confirms that hiking PL90G content from 1% to 5% aggrandizes the formulation’s VS in all cases (i.e., directly commensurate) (Table 1). Formulations F5 and F12, which had the most phospholipid, had higher vesicle diameters than formulations F13 and F4, which contained less phospholipid. Disparate works with different vesicular compositions yielded comparable results [35,41].

The experimental findings revealed an unfavorable association between the concentration of the cineole and Y2 (VS). The evidence confirms that the incorporation of cineole into the lipid bilayer membrane of the invasomes disrupts its integral packing, hence causing an increase in VS. Consequently, a higher ratio of terpenes stipulates an expanded area of the membrane to accommodate, forming bigger invasomes. It is evident from the data in Figure 1B that the concentration of cineole (B) has a substantial direct impact on the VS of MRZ-NVMs. In the same context, El-Tokhy et al. assessed the sizes of asenapine maleate-loaded invasomes [42]. This observation aligns with the hitherto divulged results of EE%, whereby an elevation in terpene levels resulted in a corresponding rise in MRZ confinement inside invasomes. As a result, the entrapment of a higher amount of lipophilic medicine inside the invasomes influences VS [22]. It should be highlighted that F8 and F9 have small vesicle sizes and demonstrated the greatest ethanol percentage levels. This might be due to ethanol’s hydrocarbon chain interpenetration into the vesicular lipid bilayers, which was hypothesized to slightly diminish the vesicle membrane’s thickness. It also promotes steric stability, which can ultimately result in VS diminution [43].

#### 3.1.3. Release Study Optimization

Table 1 and Supplementary Figure S1 showed that the different created NVM formulations increased the release of MRZ in a sustained pattern (from 53.54 ± 2.84 to 87.27 ± 2.7% over 12 h) compared to the MRZ suspension, which only released 52.19% of the original drug over the same period, contributing to its vast lipophilicity. This outcome was consistent with earlier MRZ research [44,45]. The inclusion of ethanol, which functions as a cosolvent, might explain the observed increase in drug release from MRZ-NVMs. Furthermore, ethanol increases the malleability of invasomes by fluidizing the phospholipid bilayer, straining the elastic vesicles through holes considerably smaller than their diameter [46]. Despite the notion that the release of MRZ from the designed PLGA microparticles and solid lipid nanoparticles after a duration of 48 h was found to be 79.7% and 61%, respectively, the findings of this study prove that the MRZ-NVMs formulations exploited in our research exhibited elevated levels of CMRZR and displayed improved sustained release patterns in comparison to previous investigations [47,48].

Factor A level substantially adversely impacts the %CMRZR, as the negative sign declares (*p* < 0.0001). F1 with 5% PL90G and F8 with 1% PL90G had %CMRZR values of 68.36% and 87.27%, respictively. This verdict does not come as a peculiar. This depressive effect could be attributed to the formation of extended cylinder-like micelles at rising levels of A, resulting in the forming of a network of structures that maintained the drug. That is why the quantity of MRZ accessible to diffusion diminished [49]. The impact of B on this reaction was significant, as higher concentrations of cineole led to a decrease in the proportion of %CMRZR. The observed high lipophilicity shown at elevated terpene concentrations may impede the release of the lipophilic medicine by creating a lipophilic matrix inside the invasomes. In their study on assessing propranolol hydrochloride invasomes, Teaima et al. reported similar outcomes [50]. Specifically, they noticed that invasomes containing 0.5% limonene exhibited a higher release percentage than invasomes containing 1% limonene.

The VS of MRZ was shown to have a substantial influence on the release of the drug, as supported by an additional scientific rationale. The rate of release for small-diameter vesicles is greater relative to bigger ones [51]. A linear regression approach was implemented to evaluate the MRZ release findings and ascertain the sequential pattern of MRZ-NVMs releases. Diverse NVM formulations have shown adherence to Higuchi’s diffusion-sustained pattern, which serves as the predominant release type for almost all nano-platforms. This adherence is contingent upon the coefficient, with a maximum value of R^2^ (Supplementary Table S2).

#### 3.1.4. Ex Vivo Permeation Optimization

Ex vivo permeation analysis is a potent approach to anticipating and evaluating in vivo competence of drug delivery mechanisms [52]. Transcellular permeability seems to be the predominant method of drug diffusion through the rectal mucosa, and the rectum epithelial cells are similar to that of the upper part of the gut [53]. Based on the aforementioned equation (Equation (4)), augmenting the concentration of PL90G in the invasomal formulation elevated the rectal flow of MRZ. The finding is further supported by the 3D graph, as seen in Figure 1D. The study’s results revealed that formulations F1 and F5, which had a PL90G concentration of 5% *w*/*v*, had an elevated MRZ rectal flux compared to formulations F8 and F13, which contained 1% *w*/*v* of PL90G (Table 2). The other independent variables, B and C, also exhibit a positive coefficient, indicating a synergistic effect on the response variable Y4. The data in the table demonstrate higher values of Q24 in the presence of high levels of cineole in formulations F5, F7, and F13. Similarly, the composition formulations F15, F14, and F10 exhibit the same pattern, except for the lowest levels of cineole and the observed lower Q24. The same will be witnessed when dissecting the data regarding the effect of ethanol. The lowest flux values reside in formulations that lack ethanol. For instance, F1 and F12 represent ethanol concentrations of 5% *v*/*v* and 0% *v*/*v*, respectively. Based on these results, the mechanism of phospholipid and ethanol penetration augmentation was investigated. Consistent with the findings of other authors, who asserted that when ethanol and phospholipids are combined, they have an additive effect on intercellular lipids, resulting in increased drug penetration, these findings support the hypothesis [54,55]. The increased capacity of invasomes to penetrate mucosal surfaces may be attributed to the presence of ethanol, which acts by fluidizing and disrupting the bilayer structure of the intercellular lipid matrix. Touitou et al. [56] substantiated this assertion by creating ethosomes, which are lipid-based vesicles comprising high concentrations of ethanol (>30%), phospholipids, and water. These ethosomes have shown the ability to effectively transport therapeutic agents to the deeper layers of the epidermis and the systemic circulation [57]. Terpenes are regarded as potent penetration enhancers because (1) they disrupt the hydrogen bonds that traverse the surface of the mucosa. (2) They have been demonstrated to enhance the percutaneous absorption of hydrophilic and lipophilic drugs [38]. (3) Terpenes render the particles malleable, enhancing particle deformability and a disordered bilayer lipid structure [58]. Based on the above traits, it is theorized that these distinct NVMs exhibit an amalgam of elastic vesicle properties and vesicles harboring penetration enhancers.

#### 3.1.5. Design Optimization

Regarding design optimization, the R^2^ value was computed to assess the model’s effectiveness in predicting the response variable. The adjusted and anticipated R^2^ values should be approximately within a range of 0.20 of each other to maintain logical consistency. The predicted R^2^ values demonstrated noteworthy concurrence with the anticipated R^2^ values in recorded responses [59]. This finding supports the credibility of the optimization approach and emphasizes the veracity and reliability of the proposed mathematical models for estimating the dependent variables. Therefore, this formulation was elected for additional investigation.

### 3.2. Characterization for MRZ-NVM Optimized Formulation

TEM analysis is a valuable method for conveying the findings of dynamic light scattering (DLS) in tandem with vesicle size as well as investigating the morphological characteristics of colloidal platforms. TEM analysis demonstrated the optimized MRZ-NVMs formulation as ebony specks, indicating vesicles with spherical shapes and sleek exteriors devoid of any conglomeration of MRZ particles, as seen in Figure 2. The image indicates the presence of vesicles at the same nanometer scale, suggesting a potential correlation with the VS as evaluated by the DLS approach. ZP indicates the stability of NVMs, as it shows the propensity of nano-paradigms to undergo agglomeration. The substantial magnitude of the negative results may be attributed to the involvement of ethanol and PL90G. The presence of ethanol modifies the overall surface charge, increasing vesicular charges’ stability via electrostatic repulsion, hence restricting their eventual aggregation [60]. In a similar manner, the phosphatidyl group of PL90G, which carries a negative charge, may be oriented towards the outer region, while the positively charged choline group could tilt towards the inner region. This arrangement would lead to a substantial magnitude of negative charges [61]. DSC and FT-IR results suggest that the components of the MRZ-NVMs optimized formulation are compatible, confirming no physical interactions were recorded; this is complementary to the physical stability study results.

### 3.3. Evaluation of the MRZ-NVMs pH-Triggered Gel

Injectable hydrogels are a form of biomaterial often used for controlled medicine release. This pH-responsive hydrogel was employed to promote a sol–gel phase transition, a process where the gel is administered as a free-flowing fluid but spontaneously transforms into a semisolid hydrogel soon after delivery. Viscosity enhancers may be crucial to avoid preparation outflow by conducting rectal administration. However, increasing the viscosity of the rectal formulation has detrimental consequences since it is arduous to apply high-viscosity formulations with ease and consistency. Utilizing a mucoadhesive in situ gelling system is an attractive approach to sustain mucosal contact and extend the duration of drug release [62]. The rectal mucosa exhibits tolerance to pH levels within the range of 6.8 to 7.4. Consequently, the goal was to develop formulations exhibiting gelation at pH levels within this specified range [11]. The sol–gel transition pH and time indicate that the formulation will undergo gelation at a pH level consistent with the rectum’s physiological conditions. The formulation’s rapid gelation time implies it might gel soon after rectal injection, minimizing the dose seeping from the anus. The gel spreadability is also significant for the gel’s performance, which might be related to expanding the available surface area for MRZ availability at the injection site. The bioadhesive force was sufficient to prohibit the gel formulation from obtaining access to the end of the colon, the place where the process of first-pass begins. As a repercussion, the mucoadhesive strength of the polymers in the formulation determines their residence duration. Polymers with numerous hydrophilic functional groups interact with the mucosal surface through electrostatic and hydrophobic interactions and hydrogen bonding [63]. As previously discussed in the existing literature, the use of CH/GMO, due to its bioadhesive properties, can enhance the therapeutic effectiveness of the dosage form by prolonging the length of contact at the targeted site of action [18]. Because the rectum is continuous and in a static state in comparison to the upper gastrointestinal tract, the rate of shear is thought to be modest, and the maximum viscosity of the formulation will stay steady after administration, reducing gel leakage from the administration site [12].

The aforementioned in vitro release and ex vivo diffusion perspective arises from the phenomenon known as the “dual reservoir effect,” whereby vesicles are integrated into a framework of gel to serve as reservoirs, hence impeding the diffusion of drugs from the gel [64]. Furthermore, the viscosity of the formulation impacted the quantity of MRZ released or penetrated. The gel’s high viscosity obstructs the extramicellar aqueous channels [65]. The permeation variables of the MRZ-NVMs in situ gel, on the other hand, were significantly much greater than those of the unbound MRZ gel (Table 2), which contributes to the previously described permeation-enhancers effect on the incorporated invasomal gel.

### 3.4. Pharmacodynamics Study and Behavioral Analysis

Serotonin and norepinephrine inadequacy is a clinical neurological sign of depression that manifests in impaired functionality and contentment, reluctance to move, and increased tardy behavior. Researchers scrutinized rat mobility to uncover medication effects, establish the fundamental roles of diverse neuronal structures, and screen for substances with potential psychoactivity [66]. Ensuing MRZ-NVM treatment, a substantial spike in behavioral activity was attributed to neurotransmitter restoration. MRZ promotes levels of norepinephrine and serotonin in the rat hypothalamus and cerebral cortex by combating their intake by the presynaptic neuron, allowing their availability in the synaptic gap for an extended duration. As a result, they are bound continuously to the receptors of postsynaptic neurons, causing physiological consequences associated with them.

FST is a rat behavioral assay used in melancholy inquiry, specifically to evaluate acute antidepressants, as declared in the literature. Antidepressants have long been potent in alleviating immobility, which manifests as behavioral anguish [67]. Multiple earlier studies have indicated that heightened climbing and swimming behaviors are merely in response to antidepressant medication-induced neurotransmitter levels involving norepinephrine, serotonin, and dopamine [68]. Anxiety-Based Test (ABT) was used to supplement the evaluation of depressed symptoms. A lengthy delay in digesting the repast was regarded as symptomatic of anxious conduct [69].

### 3.5. Toxicity, Histopathological, and Immunohistochemistry Analysis

The toxicity study barely shows that the optimized formulation poses no harm. In addition, liver and renal function and enzyme levels did not rise, indicating no detrimental consequences (Supplementary Figure S7).

The normal group exhibited average glial cells and neurons in the fibrillary backdrop of the white matter. Furthermore, the characteristics of the dentate gyrus, inter-neuron area, pyramidal neurons, granule cells, and blood vessels inside the cerebral cortex of the control group were typical. In the positive group (B) (Supplementary Figure S8), there are observed congested intra-cerebral blood vessels (arrow), scattered deteriorated neurons with intra-cytoplasmic vacuoles (arrow), and degenerated glial cells in a background of fibrillary cells with significant micro-cyst development (arrow). Additionally, the average inter-neuron area is also present (indicated by the arrow in B). The degenerative alterations seen in the group that received rectal administration of MRZ-NMV gel (E) were no longer present. Furthermore, the hippocampus of the MRZ-NMVs gel demonstrated a typical composition (Supplementary Figure S9), including average quantities of granule cells, pyramidal neurons, and blood vessels. The administration of MRZ-NMV gel treatment collectively improved the histological abnormalities seen in the depressed group.

Immunohistochemistry analysis evaluates the precise spot of BDNF protein expression in the cerebral cortex and the hippocampal area and identifies particular brain regions that control behaviors during FST [70]. Immunohistochemistry labeling against the BDNF protein allows for acquiring data pertaining to neuronal networks involved in the examined effects across different experimental conditions. A malfunction or a decline in BDNF may result in impaired plasticity of synapses, dwindled excitatory neurons, and increased glutamate levels, all of which may trigger depression. In Figure 7 and Figure 8, the positive control groups did not exhibit any observed immune reaction, whereas group E (rectally treated group with the optimized MRZ-NVMs in situ gel) demonstrated the highest positive reaction to BDNF compared to the negative control group.

### 3.6. Pharmacokinetics Studies

On Wister male rats, a comparative PK and brain distribution evaluation of oral MRZ suspension, free MRZ in situ gel, and MRZ-NVMs in situ rectal gel formulations was performed to explore the pharmacokinetic properties of the ameliorated formulation as well as demonstrate an affiliation with the PD study findings. The higher residence time for the optimized formulation might be attributed to the combined protracted impact caused by both the vesicular and cohesive systems [71]. The mucoadhesive functioning of CH/GMO may have restricted the gel form from reaching the upper part of the hemorrhoidal vein that drains into the mesenteric veins with the portal hepatic veins, hence the decreased lower rectum persistence. Uptake via the lower hemorrhoidal vein, where the medication enters the systemic circulation immediately, could escalate MRZ bioavailability [72]. Indeed, MRZ levels in the brain after rectal delivery of the tailored MRZ-NVMs in situ gel are considerably higher at almost all periods when compared to oral MRZ, showing the enhanced effect of the invasomal pH-responsive gel for rectal MRZ administration.

Rectal administration is a secondary route of drug administration after oral and intravenous (IV) routes. It has several advantages, including the retention of large volumes, the instant absorption of low-molecular-weight drugs, the bypassing of first-pass metabolism, controlled drug delivery, lymphatic absorption, greater effectiveness of targeted therapy, improved assimilation, and the ability to administer pharmacological agents for gastric instability. Under specific circumstances, such as vomiting, nausea, a disagreeable taste, and instances of unconsciousness occurring during post-operative interventions, the rectal route is prioritized as the primary option for patients experiencing motility difficulties in the gastrointestinal tract or in cases where there is an inflamed site of intramuscular administration [73]. Additionally, owing to invasomes components acting as permeability enhancers as well as the benefits of in situ gelling systems over conventional rectal dosage forms, a fusion of invasomes with membrane lipids boosts drug release. Dsouza et al. also reported similar findings in the literature [74]. The optimal formula allows a lower therapeutic dose to achieve a similar clinical benefit with fewer side effects. The PK characteristics obtained from the in vivo investigations demonstrate the capacity of the MRZ-loaded invasomal in situ gel to function as a drug delivery carrier. As a result, the combination of invasomal in situ gel and rectal route promoted predictable and increased MRZ plasma and brain biodistribution.

## 4. Materials and Methods

### 4.1. Materials

Mirtazapine (MRZ) was acquired from MASH Premiere (Cairo, Egypt) as a presented sample; Phospholipon 90 G (PL90G) was graciously bestowed to us by the esteemed Lipoid GmbH (Nattermannallee of Germany). Cholesterol, Ethanol, Cineole, CH/GMO, and acetonitrile (HPLC grade) were acquired from the esteemed establishment of Sigma-Aldrich (St. Louis, MO, USA). Methanol and chloroform were produced by El Nasr Pharmaceutical Chemicals Company, Cairo, Egypt. Rabbit BDNF polyclonal Antibody (Novusbio, Cat.No. #NB100-98682, Dil.: 1:500). Avidin biotin–peroxidase combination (ABC) (Vectastain ABC-HRP kit, Vector Laboratories, Inc., Burlingame, CA, USA). Sigma produces carbamazepine and Diaminobenzidine (DAB). The remaining chemicals and dissolvents used were of impeccable analytical quality. The investigation was conducted using only double-deionized water.

### 4.2. Experimental Design

Based on the preceding literature and screening trials, the causative variables and their encrypted levels (modest, middle, and elevated) were assigned for this assessment. PL90G levels (1, 3, and 5% *w*/*v*) (A), cineole % (0.5, 1, and 1.5% *v*/*v*) (B), and ethanol levels (0, 3, and 5% *v*/*v*) (C) were assigned as independent variables. The dependent responses were entrapment efficiency (EE%, Y1), nano-invasomal vesicle size (VS, Y2), the cumulative amount of MRZ released (%CMRZR, Y3), and the cumulative MRZ penetrated per unit area after 24 h (Q24, Y4). The three-level with three-factor (3^3^) Box–Behnken model was built with Design-Expert^®^ software (version 12.0.3.0, Stat-Ease Inc., Minneapolis, MN, USA) and used to create fifteen runs. The components of each run of the generated NVMs, as well as their observed responses, are shown in Table 1. To achieve the highest prediction power, three arithmetical polynomial models were examined: linear models, which merely include the main effects; 2-factor interaction models, which take into account both the effects and relations; and quadratic models, which incorporate impacts, relations, and quadratic elements. Utilizing the most appropriate model, the relevant polynomial equation was constructed and applied for each response to analyze the effect of the causative variables.

Each model’s polynomial equations were also obtained and defined as follows:

R = b0 + b1A + b2B + b3C + b12AB + b13AC + b23BC + b11A^2^ + b22B^2^ + b33C^2^ (5)

R and b0 emblematize the chosen response and the intercept, respectively. A, B, and C are the defined factors that have been inquired about, and the coefficients of regression derived from the R values are b1 to b33. The interaction indices are AB, AC, and BC, while the quadratic terms are A^2^, B^2^, and C^2^. Interacting and three-dimensional layouts (3-D) were yielded for the four reactions to investigate the interactions between the causal components.

### 4.3. Fabrication of MRZ-NVMs

The thin film hydration process was adopted to create MRZ-NVMs. Precisely weighed amounts of PL90G, cholesterol, and MRZ were dissolved in 10 mL of a chloroform/methanol mix (2:1 *v*/*v*) in a round bottom flask, pursued by the addition and dissolution of cineole. All formulations contained fixed quantities of cholesterol and MRZ (20 mg and 10 mg, correspondingly). The resulting translucent organic mixture was evaporated under a vacuum rotating at 100 rpm at 40 °C for 15 min (Stuart rotary evaporator, RE300, Wolf temperature laboratories, North Yorkshire, UK, with Stuart vacuum pump, RE3022C, Wolf Laboratories, North Yorkshire, UK) to achieve a thin, wholly homogeneous film with no solvent residues upon the flask inner surface. An ethanolic–phosphate buffer solution mixture was prepared for the hydration step: 10 mL (PBS) (PH 7.4) containing different ratios of ethanol by rotation at 70 rpm for one h. Invasomal dispersions were also sonicated in bath sonication (Sonix TV ss-series ultrasonicator, North Charleston, SC, USA) for 5 min to produce nano-sized suspended formulations, which were then stored at four °C until the purpose [31,75].

### 4.4. Characterization of MRZ-NVMs

#### 4.4.1. Determination of MRZ Encapsulation Percent

MRZ entrapment was estimated indirectly (based on free MRZ) [76]. Centrifugation separated the unentrapped MRZ from the generated MRZ-NVMs dispersion milieu for 2 h at 22,000 rpm and 4 °C in a cooling centrifuge (SIGMA 3-30K, Steinheim, Germany). The supernatant underwent filtration with a pore size of 0.45 µm Millipore polycarbonate membrane filter (Whatman Ltd., Springfield Mill, UK). A UV spectrophotometer (JASCO V530, Tokyo, Japan) was used to assay absorbance at 289 nm [77]. Measurements have been recorded in three separate instances. Equation (6) was used for assessing the percentage of MRZ entrapped, as shown below:

(6) $$EE\%=\left(\frac{\mathrm{MRZ}\;\mathrm{total}\;\mathrm{amount}\;-\;\mathrm{unentrapped}\;\mathrm{MRZ}}{\mathrm{MRZ}\;\mathrm{total}\;\mathrm{amount}}\right)\times 100$$

#### 4.4.2. Determination of Vesicle Size (VS)

The mean diameters of MRZ-NVMs and polydispersity index (PDI) were evaluated using a Malvern Zeta Sizer (Malvern Instruments, Malvern, UK) and the dynamic light scattering approach (DLS) at 25 ± 2 °C. The recently prepared nanosuspensions had a dilution process at a ratio of 1:10 in deionized water prior to testing. The angle of scattering was fixed at 90 degrees. Each formulation picked up three determinations [78].

#### 4.4.3. In Vitro Release Examination of MRZ from MRZ-NVMs

A refined Franz diffusion cell with a 6.15 cm^2^ operating diffusion area was employed to measure MRZ release from various invasomal formulations and MRZ suspension (USP dissolution tester apparatus, type 1, Hanson Research, SR 8 Plus model, Chatsworth, CA, USA). The donor section of the Franz cell was laden with an invasomal nanosuspension (different volumes of the nano-formulation containing a drug amount equal to 3 mg). The drug was then penetrated by a dialyzing cellulose barrier (Mw cutoff = 12,000 Da) that had been maintained overnight in the buffer prior to the launch of the release investigation. The receptor chamber was filled with 50 mL of PBS with pH 7.4 (composition: 8 g of NaCl, 1.44 g of Na_2_HPO_4_, 0.2 g of KCL, and 0.24 g of KH_2_PO_4_ dissolved in one liter of distilled water) was supplied. Moreover, 0.1% (*v*/*v*) of polysorbate 80 was added to the receptor chamber to maintain the sink condition of MRZ concerning solubility studies [6]. Throughout the dissolution investigation, the receptacle chamber was stirred at a speed of 100 rpm, and the temperature was fixed at 37 ± 0.5 °C [78]. Samples of 2 mL volume were obtained from each releasing chamber at regular intervals (0.5, 1, 2, 3, 4, 6, 8, and 12 h). Every time a sample was taken, the eliminated volume was promptly replaced with an equiponderant volume of untainted medium to ensure a consistent volume (sink conditions). The MRZ concentration was tested using spectrophotometry at a wavelength of 289 nm. According to the used, validated spectrophotometric method for the determination of MRZ [79], the resulting linearity range of MRZ was (5–40 µg/mL), and the accuracy % recovery was 99.12 ± 0.43 with %R.S.D of 1.30%. Moreover, the LOQ and the LOD values were 1 µg/mL and 0.2 µg/mL, respectively. The release experiment was repeated three times, and the mean of the three readings was calculated. Equation (7) was used to calculate the percentage of CMRZR.

(7) $$\mathrm{CMRZR}\;(\%)\;=\;\%\mathrm{release}\;\mathrm{at}\;\mathrm{time}\;t\;+\left(\frac{\mathrm{Sample}\;\mathrm{volume}\;\mathrm{withdrawn}}{\mathrm{total}\;\mathrm{media}\;\mathrm{volume}}\right)\times \;\%\;\mathrm{released}\;\mathrm{previously}\;(t-1)$$

Fitting the observed release data to zero-order, first-order, and Higuchi diffusion models yielded the MRZ release kinetics. The model that best fits the MRZ release mechanism was chosen based on the model with the highest correlation coefficient value (R^2^) [80].

#### 4.4.4. Ex Vivo Permeability Investigation

The efflux of MRZ from the produced nano-invasomal formulations and MRZ suspension was investigated using a Franz diffusion cell with a working area of 6.15 cm^2^. A freshly dissected cow rectum was employed as a permeation model barrier, which was maintained in PBS (pH 7.4) for 1 h before the study to equilibrate. A volume of 50 mL of PBS (PH 7.4) was utilized to fill the receptor section, perpetually stirred at a velocity of 100 rpm for 24 h, and maintained at a thermostatic temperature of 37 ± 0.5 °C. 0.1% (*v*/*v*) polysorbate 80 was added to the receptor chamber to maintain the drug sink condition. Salem et al. previously reported on all of the procedures employed in this study [71]. Specific amounts of MRZ-NVMs suspensions (equal to 3 mg of MRZ) were inducted into the donor section of the Franz cells to be in contact immediately with the rectal mucosa. At 0.5, 1, 2, 4, 6, 8, 12, and 24 h, a 2 mL sample was drained from the receptor chamber, and the withdrawal vehicle was rapidly replaced with an entirely fresh buffer of an equivalent volume at every interval to maintain sink requisites. To determine MRZ content, filtered and adequately diluted test samples were spectrophotometrically analyzed at 289 nm. The total amount of MRZ permeated the cow rectum tissue was determined for each time interval using Equation (8) [81].

(8) $$\mathrm{Cumulative}\;\mathrm{amount}\;=\;Vol1\;\times \;Ct\;+\;\left[Vol2(\sum C1\;+\;\cdots +\;Ct\;-\;1)\right]$$

Vol1 is the receptor compartment volume (50 mL), Vol2 is the volume collected at each interval (2 mL), and Ct is the sample concentration at time n.

The experiment established three estimations for every formulation (three separated instances), and the data were reported as the average ± SD. For each formulation, MRZ’s permeability coefficient Kp (cm/h) was computed with the division Jss (µg/cm^2^/h) by the commencing MRZ concentration uploaded in the donor section. The slope of the straight line was used to calculate the MRZ steady-state flux (Jss) (μg/cm^2^/h) [78]. For each formulation, the permeability coefficient Kp (cm/h) of MRZ was computed with the division Jss (µg/cm^2^/h) by the commencing MRZ concentration uploaded in the donor section. The MRZ time to permeation start (lag time in minutes) was calculated using the x-intercept of the rectilinear section [82]. The enhancement ratio (ER) was also calculated to examine the effectiveness of nano-invasomes in increasing MRZ permeability when contrasted to MRZ suspension. The following equation (Equation (9)) was used to calculate ER:

(9) $$\mathrm{Enhancement}\;\mathrm{ratio}\;(\mathrm{ER})\;=\;\frac{\mathrm{Kp}\;\mathrm{of}\;\mathrm{the}\;\mathrm{MRZ}\;-\;\mathrm{NVMS}\;\mathrm{formulation}}{\mathrm{Kp}\;\mathrm{of}\;\mathrm{the}\;\mathrm{control}\;\mathrm{MRZ}\;\mathrm{suspension}}$$

### 4.5. Evaluation and Optimization of MRZ-NVMs

#### 4.5.1. Experimental Model Evaluation

Proving the ANOVA test at *p* < 0.05, design-expert software was adopted to examine all quantitative outcomes from Y1 through Y4. It includes a model matrix for appraising the most suitable model and polynomial formulas with mathematical correlations among autonomous variables. The Design-Expert software’s point prediction approach was used to pinpoint the optimum formulation and entrench its ability to establish the independent variable levels (A, B, and C), obtaining the smallest NVMs-VS connected with the apical EE%, % CMRZR, and Q24. The optimal composition was then exposed for further investigation.

#### 4.5.2. Characterization of the Optimized MRZ-NVMs Formulation

The optimized formulation was created and tested in various tests, including EE%, VS, transmission electron microscopy (TEM), zeta potential (ZP), and stability. The optimized formulation in vitro release kinetics and ex vivo permeation pattern investigation were further imposed using the same technique and procedures provided previously. Also, differential scanning calorimetry (DSC) and Fourier-transform infrared spectroscopy (FT-IR) analysis have been investigated.

#### 4.5.3. Morphological Examination by TEM

The morphology of MRZ-NVMs was examined using a transmission electron microscope (JEM-1400, Jeol, Tokyo, Japan). One drop of the optimal NVM formulation was laid on a copper grid, and the surplus was removed with filter paper. Following that, a drop of 2% *w*/*v* phosphotungstic acid (negative staining) was applied, and the excess was wiped. The air-dried samples were ultimately examined using a TEM at 80 kV [83].

#### 4.5.4. Zeta Potential

The optimum nano-invasomal dispersion’s zeta potential (ZP) was reported with a Malvern Zeta Sizer (Malvern, UK), and the average of three measurements (*n* = 3) was computed. The speed of the vesicles via a liquid was ascertained using an electrophoresis-based method after introducing an electrical field.

#### 4.5.5. Stability Study

For three months, the optimum MRZ-NVMS formulation was stored in a hermetically sealed glass vial at 4 °C. After 30, 60, and 90 days, collected samples from the optimized formulation were characterized by measuring their ZP, VS, and EE% and contrasted with the recently concocted one to evaluate its physical reliability of the optimal formulation [84]. The records were iterated thrice.

#### 4.5.6. Differential Scanning Calorimetric Analysis

The DSC assay was applied to assess the drug’s thermal behavior both alone and in combinations with all of the utilized invasomal components with a differential scanning calorimeter (Shimadzu Corporation, Kyoto, Japan). Two- to four-milligram samples were placed on metal pans in a nitrogen atmosphere and heated at a 10 °C/min rate from 25 to 250 °C [18].

#### 4.5.7. Fourier-Transform Infrared Spectroscopy Analysis

FTIR (8400s, Shimadzu, Japan) was used to study the chemical interactions and crystallinity of MRZ, cholesterol, PL90G, cineole, and ethanol. Both MRZ alone and in a mixture with the other ingredients were studied based on the distinctive MRZ peaks, whether loading into NVMs causes peaks to shift and mask or new peaks emerge. Preceding the evaluation of samples from 500 to 4500 cm^−1^, they were carefully pulverized and combined with KBr [1].

#### 4.5.8. Elaboration of MRZ-NVMS pH-Sensitive In Situ Gel

Gelling systems containing variant chunks of CH/GMO were tested for gelling capacity in order to identify content convenient for use as an in situ gelling system. In pursuance of assembly, the in situ pH-triggered MRZ-NVMs gel outlined by Ganguly et al. Concisely, CH was dissolved in 0.33 M citric acid to achieve a concentration of 0.67% *w*/*v*, and then GMO (0.27% *w*/*v*) was melted at 45 °C and incorporated into the CH solution while being stirred continuously for 50 min. Finally, 30 min of sonication was used to add the determined amount of MRZ-NVMs to the CH/GMO solution [18]. For comparison, an in situ plain gel of free MRZ was further synthesized using an identical procedure.

### 4.6. Characterization of MRZ-NVMS In Situ Gelling System

The structural characteristics (texture and transparency) of the freshly manufactured MRZ-NVMS gel were examined visually. A formerly described procedure was used to analyze the in situ gel’s release and permeation.

#### 4.6.1. Measurement of Sol–Gel Transition pH, Gelation Time, and Spreadability

pH-responsive in situ gels are distinguished by their capacity to gel at a definite physiological pH. The pH of formulations was adjusted with a concentrated NaOH solution to determine the sol–gel transition pH. The addition of NaOH solution (60 μL, 12 M) was able to generate hydrogel in <60 s [85]. Using a pH meter, adjust the pH until gelling is achieved. The formulation was evaluated for gelation, which was said to have occurred when the formulation’s meniscus would no longer move when tilted through 90 °C [86]. The time of the first gelling detection was defined as gelation time. Three successive measurements were taken.

The spreadability of the gel was evaluated by employing a simple technique. In brief, 0.5 grams of the gel was dropped on a glass plate with a specified mark, which was later overlaid with a second one. A 500 g weight was placed on the top plate and was steady for 5 min, with no additional spreading projected. The spread-out circle around the mark was measured in diameter.

#### 4.6.2. Drug Content and In Vitro Mucoadhesion Measurement

A total of 0.1 gram of the gel preparation was obtained and subjected to sonication in 5 milliliters of ethanol to induce vesicle breakdown. The MRZ concentration was measured spectrophotometrically at 289 nm. The mucoadhesive strength of the gel formulation was assessed using a modified balancing technique to determine the force required to dislodge it from rectal mucosal tissue. Cyanoacrylate glue was used to bind a one-centimeter slice of rectal mucosa to two glass surfaces. The first slide was coated with 50 mg of gel and placed under the height-adjustable pan. On the bottom of the same pan, a different slide containing a mucosal segment was mounted in an inverted manner. To establish close contact, the two slides with the gel composition between them were maintained in contact for two minutes. Then, the weight in the second pan continued to rise until the slides separated from one another. The least weight required to separate the mucosal tissue from the formulation surface was used to calculate the mucoadhesive force, which is given as the detachment stress in Dynes/cm^2^ [87]. The mucoadhesive force was calculated as follows:

(10) $$\mathrm{Mucoadhesive}\;\mathrm{strength}\;(\mathrm{Dynes}/{\mathrm{cm}}^{2})\;=\;\frac{m\;\times \;g}{A}$$

where m is the weight required to separate two slides in grams, g is gravity’s acceleration (980 cm/s^2^), and A is the area of rectal mucosa.

#### 4.6.3. Rheological Property Determination

The rheological parameters of the optimized MRZ-NVMs gel prior to and subsequent to gelation were investigated using a cone and plate viscometer (Brookfield DV-III, spindle 52, Middleboro, MA, USA). The investigation of the pH dependence of formulation viscosity included measuring the viscosity of formulations at pH of 4.0 and 7.4 and shear rates ranging from 20 to 200 (s^−1^) [88,89]. A trapezoidal rule was used to reveal the area of hysteresis loops. Using Farrow’s equation (Equation (11)), the flow behavior of the gel formulation was investigated as follows:

(11) Log G = N Log F − Log Ƞ

where N is Farrow’s constant, η is the viscosity (cp), G is the shear rate (s^−1^), and F is the shear stress (dyne/cm ^2^).

### 4.7. In Vivo Studies

#### 4.7.1. Ethical Approval and Pharmacodynamic Study

Studies of pharmacokinetics (PK) and pharmacodynamics (PD) were carried out based on the ethical norms of Beni-Suef University’s committee and the Helsinki protocols. The institutionalized care of animals sanctioned all animal manipulation techniques and use committee at the Faculty of Veterinary Medicine at Egypt’s Beni-Suef University (approval code: BSU-IACUC 022-438).

The antidepressant effectiveness of MRZ-NMVs gel has been assessed on 30 robust adult male Wistar rats weighing between 160 and 180 g, assigned to five distinct groups of six animals each. Antecedently experiencing the affliction of depression inducement or the empirical methodologies, rats had been habituated to a conventional rodent diet for seven days right after their advent. The rodents had a supply of new, potable water throughout the day. Evading feces, rats that were given the rectal formulation were starved for 16 h before administering the dose and merely received water.

#### 4.7.2. Induction of Depression and Experimental Protocol

In this animal approach, the forced swimming test (FST) was employed to provoke depressive-like symptoms. Apart from the standard monitoring group, all animals were compelled to swim underwater inside a cylindrical container filled with water, with a height of 50 cm and a diameter of 22 cm (35 cm depth) (temperature: 25 ± 2 °C). Rats were treated to daily swim workouts for seven days using the procedures outlined by Kaur et al. [67]. After each swimming session, the rats were hauled out of the water, dried with bedding, and eventually returned to their separate cages. They had a 15 min pre-examination swim time on their initial day, ahead of a 5 min swim period on the subsequent day into two distinct periods: night and day bouts. In G1, rats exhibited no depressive induction, serving as the negative control group; however, in G2, rats were supplied with 0.5 milliliters of isotonic saline solution as a positive monitoring for depressed animals. G3 received an oral dose of MRZ suspension (2.5 mg/mL; 15 mg/kg). G4 was dosed rectally with 400 μL of the free MRZ pH-sensitive in situ gel formulation (15 mg/kg). G5 received the same dose as group 4 and route, with the exception of the optimized MRZ-NVMs pH-sensitive in situ gel formulation. MRZ suspension was prepared using carboxy methyl cellulose (0.5% *w*/*v*) to suspend MRZ in well-purified water. A 16-gauge feeding needle with a ball tip and a syringe were used to provide oral doses. A synthetic syringe coupled to a needle was used to insert gel over the anus at 4 cm depth for intrarectal dosage. For around 10 min, the rats were held in a ventral position with their heads low, and the rectum closed until the drug had dissipated entirely. A collection of tests, such as the Forced Swim Test (FST), Tail Suspension Test (TST), Sucrose Preference Test (SPT), Open Field Test (OFT), Social Interaction Test (SIT), and Anxiety-Based Test (ABT), were all implemented in a series of behavioral assessments.

#### 4.7.3. FST Immobility, Swimming, and Climbing, Tail Suspension Test (TST), and Sucrose Preference Test (SPT)

The rats were stuck in a keg brimming with water for the mandated swim testing. After a 10 min dosage, the activities of immobility, soaring, and swimming periods were carefully detected. The rat appeared immobile upon its combat cessation and surrender, buoyed in the water [90]. After each swim session, the water tank was methodically emptied and replaced to minimize any possible altered behavior attributed to contamination.

Rats were hanged by the ends of their tails from elevated bars 50 cm high over the surface using sticky tape. A climbing spigot was fitted to refrain the rat from grasping its tail and ascending. The time of immobility and absence of escape attempts for a period of 6 min, displayed by each rat 30 min after drug treatment, were tracked and evaluated [91]. In the sucrose-based predilection test, a decreased affection for sweet foods reflects misery, which may be rectified with long-term antidepressant therapy.

The rats were fed several food items from day one through the fourth day of the test. On the first two days, the rats received two bottles of pure water; on the third day, they were supplied with two bottles of sucrose (1%); and on the fourth day, they received a bottle of clean water and a bottle of sucrose (1%) [92]. After 12 h of water and food starvation, each rat received 200 mL of each 1% sucrose solution and clean water. The quantity used for both was reckoned, and sucrose preference was estimated using the following arithmetic (Equation (12)):

(12) $$SP\;\left(\%\right)\;=\;\left[\frac{sucrose\;solution\;consumption\;\left(g\right)}{total\;consumption\;\left(g\right)}\right]\;\times \;100$$

#### 4.7.4. Open Field Test, Social Interaction Test (SIT), and Anxiety-Based Test (ABT)

In OFT, the rats were allowed to explore their spaces for 6 min after being put in a 100 × 50 cm black cube enclosure; during the first half-minute, rats acclimate to the surroundings. Throughout the test period, camera video was used to estimate the duration elapsed in the center of the OFT box and the entire afar traversed [92].

The Social Interaction Test (SIT) employs footage recording along with evaluation to assess social grooming, creeping, snorting, wrestling, seeking out, and violent conduct in rats. Additionally, the test assesses the degree of test-novel rat interactions [93]. The tranquil room was an enclosed space where the trials were carried out (30 × 30 cm in length and breadth, with a height of 60 cm). The room was bathed in bright white light. Two rats with similar weights from the same study group were put on a diagonal in opposing parts of the testing venue for six minutes at the beginning of each testing session.

The time it takes to approach and eat food in unfamiliar surroundings after a prolonged period of food restriction is tracked for rats in this experiment (ABT). The duration it takes an animal to begin feeding exposes its behavioral approach, as described in previous publications [94,95]. The ABT test can assess anhedonia in the context of a food incentive and an unknown open environment, making it efficient for assessing depression. This is alongside the consideration that conflict resolution is adversely associated with despair and anxiety [69]. The rats’ food was confiscated from their cages after they were weighed, but they retained their water. The rats were placed in a 40 × 60 × 50 cm wooden cage designed to diminish outer aural or visual stimuli after 24 h without food. The investigated animal was confined in a restricted cage and fed a known-weight food item for 5 min. The leftover meal was weighed to determine the amount of food consumed. After a thorough checkup, all rats were restored to their original cage and allowed ample food and water [96,97].

#### 4.7.5. Toxicity, Tolerability, and Histopathological Studies

From experiment onset till euthanasia, rat weights were monitored. The research also observes mortality and aging-related changes in anticipation and manners [98]. EDTA-collected blood samples are used to examine the complete blood count (CBC) in various therapeutic scenarios. Nevertheless, serum samples are taken to determine the formula’s safety for the liver and kidneys. Many parameters were assessed, including hemoglobin (Hb), red blood cells (RBC), mean corpuscular volume (MCV), mean corpuscular hemoglobin (MCH), mean corpuscular hemoglobin concentration (MCHC), white blood cells (WBCs), platelet count, creatinine, urea, aspartate aminotransferase (AST), and alanine aminotransferase (ALT).

Histological studies on the rectal mucosa and brain were performed to assess the safety of the supplied formulation rectally and rule out potential side effects [26]. The rectal mucosa and brain tissues of the negative control, free MRZ in situ gel, and MRZ-NMVs in situ gel groups were collected from slaughtered animals that were humanely euthanized using a 1:1 mixture of ketamine (90 mg/kg) and xylazine (5 mg/kg). The rats were given a 0.1 mL/100 g b.wt. intraperitoneal injection. The mucosa was surgically excised, decalcified, and stored in a 10% buffered formalin solution for sectioning and analysis. The sections were stained with hematoxylin and eosin (H&E) and inspected under a light microscope [99,100].

#### 4.7.6. Brain-Derived Neurotropic Factor (BDNF) Immunohistochemistry Analysis

After one night of air drying at 25 °C, the brain slices were fixed in paraformaldehyde (4%) for 30 min. The slices were washed twice after fixation and incubated at 25 °C for 24 h. The avidin–biotin–peroxidase combination (ABC) mounted the paraffin slices on positively charged slides.

ABC technique reagents were added after incubating sections from each group with these antibodies. Marker expression was labeled with peroxidase and colored with DAB to detect the antigen–antibody complex. Negative controls were included using non-immune serum in place of the primary or secondary antibodies. An Olympus BX-53 microscope was used to analyze IHC-stained sections. The density of tagged nuclei was determined by quantifying them in certain anatomical locations of the brain. BDNF expression levels were assessed independently in each hemisphere for each segment thrice [101].

#### 4.7.7. Scoring of Immunohistochemistry Results and Statistical Analysis

The scoring of results is the response area% in 10 microscopic fields, and it is calculated using image J 1.53t, Wayne Rasband and authors, National Institutes of Health, USA. The experiment results, in triplicate, are shown as the mean ± standard deviation (SD). The data acquired for the behavioral study were statistically analyzed utilizing a one-way analysis of variance (ANOVA) and a least significant difference (LSD) post hoc test. SPSS version 20 was used for statistical analysis in the study. A significance level of *p* < 0.05 was assumed to be statistically significant.

### 4.8. Plasma and Brain Bioavailability Study

PK parameters of the rectal and oral formulations were assessed using 72 Wistar rats (male, 200–250 g). Three groups of 24 rats each were formed. Three rats were used for each formulation at any point in the investigation (3R approach, *n* = 3). Following an overnight period of abstaining from food, the rats were given a dose, confined for a sample, and kept conscious throughout the experiment. The oral, rectal plain gel, as well as rectal-nano-gel preparation of MRZ were administered at a fixed level of 15 mg/kg body weight to compare pharmacokinetic results [102,103]. The first group received the MRZ suspension orally (description of dosage illustrated in PD study). The second and third groups received rectally given free MRZ in situ gel and the improved MRZ-NVMs pH-responsive gel formulation, respectively. The PK studies were conducted for 12 h in the brain and 72 h in the plasma to establish a distinct elimination phase of MRZ for the study of several PK parameters. The anus was covered with adhesive tape after dosing to avoid any leakage of the provided dosage. The medication was included in any formulation at 8 mg/mL concentration.

#### 4.8.1. Collection and Analysis of Plasma and Brain Samples

Hematological samples (0.5 mL) were collected from anesthetized rats’ retro-orbital punctures (*n* = 3 per time point) at predetermined intervals (0.5, 1, 2, 4, 8, 12, 24, 48, and 72 h) after MRZ dosage in 1.5 mL microcentrifuge-heparinized tubes. Upon centrifugation at 2000 rpm for 20 min, the plasma was sectioned. In order to assess the improved MRZ-NVMs in situ gel’s capacity for brain targeting, brain samples were taken at the end of each and every sampling (at 0.5, 1, 2, 4, 6, 8, and 12 h). The animals were humanely killed by subjecting them to an overabundance of diethyl ether in a glass container tightly sealed. Their skulls were then severed, and their brains were obtained. The removed brains were promptly rinsed with saline to remove any remaining blood. After that, they were dried and homogenized in physiological saline at a ratio of 1:4 (*w*/*v*) according to their weights. The homogenization required 5 min at 20,000 rpm in a tissue homogenizer (Fisher Scientific, Dreieich, Germany) [104,105]. The serum and brain homogenates were kept at −20 °C until their MRZ content was determined.

All frozen samples were extracted utilizing a liquid extraction method. Each sample (500 µL) was treated with 0.5 mL of the internal standard carbamazepine (1000 ng/mL dissolved in acetonitrile) before MRZ was extracted with acetonitrile (5 mL) [45]. To obtain MRZ in acetonitrile, mixtures were vortexed and centrifuged for 10 min at 6000 rpm. The top layer was carefully collected and transferred to a new tube before completely evaporating. Before being placed in injection vials, the desiccated test tube section has been dissolved in 500 µL of acetonitrile. A 20 µL portion of this reconstituted extraction was provided for MRZ measurement into the HPLC system. The samples were analyzed using an HPLC system (Waters Alliance 2695 HPLC system with Waters 2996 PDA, SpectraLab Scientific Inc., Ontario, CA, USA). As a mobile phase, phosphate buffer (pH 3.9) and acetonitrile (60:40) were used, and plasma samples from rats were loaded into Kromasil C18 (Kromasil, Göteborg, Sweden) (5 m, 4.6 × 150 mm) by isocratic elution. The flow rate at room temperature was 1.5 mL per minute. The photodiode array detector detected the drug’s wavelength (λmax 289 nm) [106].

#### 4.8.2. Pharmacokinetic Analysis and Statistics

The PK of the oral and rectal formulations was investigated using a non-compartmental model in WinNonlin (edition 1.5, Scientific Consultants, Inc., Rockville, MD, USA). The highest levels of MRZ in the blood and brain (Cmax) and the time it took to reach these levels (Tmax) were calculated by plotting the average MRZ levels in blood and brain samples against the number of hours subsequent to oral and rectal administration. The program was executed to compute the half-life (the duration necessary to reach 50% of the MRZ plasma level) as well as the mean residence time (MRT). Moreover, the trapezoidal method was used to compute the area beneath the curve from 0 to t h. The brain-to-blood ratio for the rectal or oral formula was determined over time. Ultimately, the relative bioavailability (Frel) of the rectal formulation VS MRZ oral susp in plasma and the brain was found using the following method (Equation (13)):

(13) $$F\left(rel\right)=\left[\frac{AUC\;\left(MRZ\;-\;NVM\;in\;situ\;gel\right)}{AUC\left(oral\;formulation\right)}\right]\;\times \;100$$

Statistical analysis was performed to assess if there was evidence of significance in PK variables between the rectal MRZ-NVM in situ gel formulation, the oral MRZ suspension, and the free MRZ in situ rectal gel. When the level of significance was set at *p* < 0.05, the findings were determined to be statistically different. The tandard deviation (SD) generated from at least three points represented all data acquired throughout the in vivo inquiry.

## 5. Conclusions

NVMs were efficaciously developed as a malleable nano-carrier for the innovative brain targeting of MRZ through rectal delivery, with the goal of successfully managing depression in the current study. The NVM formulations were skillfully used via the thin-film hydration process. The Box–Behnken design was used to optimize the vesicular dispersions. The optimal NVM formulation exhibited a monodisperse spherical nanostructure, diminutive vesicle size, raised z potential, suitable EE%, extended release profiles, and enhanced rectal permeability for drug delivery. By means of behavioral tests and toxicological studies, immunohistochemical and histological investigations demonstrated that the administration of rectal MRZ-NVMs in situ gel led to significant improvement in depression symptoms in rats. The pharmacokinetic analysis revealed significantly enhanced bioavailability, as well as prolonged T1/2 and MRT, of the optimized MRZ-NVMs in situ gel in comparison to both the free in situ gel and oral suspension. As a result, the implications suggest that NVMs have the potential to provide non-invasive rectal delivery of MRZ while also offering a viable platform for depression treatment.

## Figures and Tables

**Table 1:** Box–Benkhen design, independent factors, and the four responses of MRZ-NVMs formulations.

**Factor Code**	**Factor Name**	**Factor Level**			**Response Code**	**Response Name**		**Unit**
		**−1**	**0**	**+1**	Y_1_	Entrapment Efficiency		(EE %)
A	PL90G concentrations (%*w*/*v*)	1	3	5	Y_2_	Invasomes vesicle size (VS)		(nm)
B	cineole percentages (%*v*/*v*)	0.5	1	1.5	Y_3_	Cumulative amount MRZ released after 12 h (CMRZR)		%
C	ethanol concentrations (%*v*/*v*)	0	3	5	Y_4_	Q_24_*		(µg/cm^2^)
**Run**	**PL90G (%*w*/*v*)**	**Cineole (%*v*/*v*** **)**	**Ethanol (%*v*/*v*** **)**		**Y_1_ (%) ± SD**	**Y_2_ (nm) ± SD**	**Y_3_ (%) ± SD**	**Y_4_ (µg/cm^2^) ± SD**
F1	5	1	5		87.59 ± 1.56	264.7 ± 11.9	68.36 ± 1.58	477.33 ± 15.31
F2 *	3	1	3		75.27 ± 2.54	207.03 ± 3.5	73.23 ± 1.95	431.42 ± 8.39
F3 *	3	1	3		74.85 ± 1.75	216.43 ± 4.3	71.91 ± 1.31	428.47 ± 9.50
F4	1	1	0		62.35 ± 1.89	130.77 ± 9.6	79.23 ± 0.57	295.68 ± 14.74
F5	5	1.5	3		97.15 ± 1.40	307.83 ± 6.2	53.54 ± 2.84	470.33 ± 16.81
F6 *	3	1	3		76.71 ± 1.28	224.17 ± 8.9	69.48 ± 0.75	421.86 ± 7.3
F7	3	1.5	0		86.91 ± 1.02	227.1 ± 6.8	68.19 ± 2.57	379.32 ± 10.7
F8	1	1	5		59.41 ± 2.18	138.57 ± 5.6	87.27 ± 2.7	331.13 ± 15.51
F9	3	0.5	5		68.32 ± 1.40	171.43 ± 5.2	73.32 ± 0.94	417.71 ± 10.69
F10	1	0.5	3		62.39 ± 3.87	88.97 ± 7.9	82.12 ± 2.25	346.51 ± 11.21
F11	3	1.5	5		81.46 ± 2.34	285.97 ± 11.0	74.18 ± 3.11	393.75 ± 14.17
F12	5	1	0		93.17 ± 3.04	267.87 ± 6.9	55.95 ± 1.80	425.34 ± 11.62
F13	1	1.5	3		71.51 ± 0.85	175.27 ± 3.6	81.52 ± 1.15	360.96 ± 19.02
F14	3	0.5	0		77.56 ± 1.72	187.9 ± 3.1	70.96 ± 2.75	327.26 ± 17.32
F15	5	0.5	3		91.27 ± 1.42	247.13 ± 7.0	64.33 ± 1.91	460.99 ± 11.06

Q_24_* = Cumulative amount of drug permeated after 24 h. SD (standard deviation of *n* = 3); all formulations contain 10 mg MRZ, * indicates center points of the design.

**Table 2:** Permeation parameters of the MRZ-NVMs formulations.

Formula Nom	Q24 (µg/cm^2^)	Flux (Jss) (µg/cm^2^·h)	Kp (cm/h)	Lag Time (min)	Enhance Factor	PDI
F1	477.33 ± 15.31	34.75 ± 2.05	0.034747 ± 0.0021	15.57 ± 2.43	2.33	0.354 ± 0.0274
F2 *	431.42 ± 8.39	25.98 ± 1.86	0.025976 ± 0.0018	13.63 ± 3.12	1.74	0.241 ± 0.0180
F3 *	428.47 ± 9.50	24.84 ± 2.14	0.024843 ± 0.0032	18.19 ± 1.94	1.67	0.345 ± 0.0053
F4	295.68 ± 14.74	16.98 ± 0.95	0.016975 ± 0.0025	17.32 ± 2.63	1.14	0.268 ± 0.0251
F5	470.33 ± 16.81	33.67 ± 1.32	0.033674 ± 0.0004	20.87 ± 2.71	2.26	0.423 ± 0.0223
F6 *	421.86 ± 7.3	23.11 ± 1.56	0.023111 ± 0.0023	23.46 ± 1.82	1.55	0.322 ± 0.0135
F7	379.32 ± 10.7	19.58 ± 0.86	0.019580 ± 0.0012	19.27 ± 3.42	1.31	0.434 ± 0.0356
F8	331.13 ± 15.51	17.98 ± 1.89	0.017984 ± 0.0071	13.69 ± 3.16	1.21	0.287 ± 0.0316
F9	417.71 ± 10.69	29.83 ± 2.43	0.029834 ± 0.0062	14.04 ± 1.86	1.99	0.339 ± 0.0307
F10	346.51 ± 11.21	21.91 ± 1.42	0.021911 ± 0.0023	17.61 ± 1.92	1.47	0.239 ± 0.0148
F11	393.75 ± 14.17	25.79 ± 0.98	0.025789 ± 0.0030	14.47 ± 1.56	1.73	0.281 ± 0.0221
F12	425.34 ± 11.62	30.86 ± 1.28	0.030863 ± 0.0086	15.46 ± 0.96	2.07	0.248 ± 0.0114
F13	360.96 ± 19.02	23.41 ± 0.56	0.023411 ± 0.0045	13.97 ± 1.21	1.57	0.293 ± 0.0264
F14	327.26 ± 17.32	17.71 ± 0.84	0.017713 ± 0.0052	22.73 ± 2.56	1.19	0.340 ± 0.0099
F15	460.99 ± 11.06	33.38 ± 1.73	0.033386 ± 0.0019	24.87 ± 2.11	2.24	0.289 ± 0.0139
MRZ susp	247.06 ± 11.81	14.92 ± 0.61	0.014919 ± 0.0014	41.29 ± 3.78	-	-
MRZ-NVMs optimum formula	468.68 ± 14.31	31.27 ± 1.34	0.031270 ± 0.0012	14.84 ± 1.30	2.09	0.252 ± 0.0162
MRZ-free gel	225.94 ± 12.63	11.40 ± 1.06	0.011401 ± 0.0007	45.73 ± 3.53	0.76	-
MRZ-NVMs in situ gel	418.55 ± 11.42	23.61 ± 2.21	0.023605 ± 0.0024	24.38 ± 2.19	1.58	-

* indicates center points of the design

**Table 3:** PK parameters for the oral MRZ, free MRZ rectal gel, and the MRZ-NVMs in situ rectal gel.

Pharmacokinetics Parameter	Oral MRZ Suspension		Rectal Free MRZ Gel		Rectal MRZ-NVMs In Situ Gel	
	Plasma	Brain	Plasma	Brain	Plasma	Brain
C_max_ (ng/mL)	972.84 ± 34.28	494.94 ± 34.21	1536.60 ± 73.16 (a)	967.55 ± 42.76 (a)	2616.99 ± 127.68 (a,b)	2064.294 ± 102.94 (a,b)
T_max_ (h)	2	2.67 ± 0.94	3.33 ± 0.94	4	4.0 (a)	4.67 ± 0.94
T_1/2_ (h)	35.01 ± 1.86	5.82 ± 0.27	40.10 ± 1.98 (a)	8.48 ± 0.31	52.94 ± 1.29 (a,b)	12.54 ± 2.53 (a)
Kel	0.0198 ± 0.001	0.1193 ± 0.0054	0.017 ± 0.0008 (a)	0.0817 ± 0.0028 (a)	0.0131 ± 0.0003 (a,b)	0.05731 ± 0.0101 (a,b)
MRT (h)	51.47 ± 2.32	8.92 ± 1.20	54.69 ± 2.78	13.46 ± 0.46	72.96 ± 2.32 (a,b)	19.62 ± 3.67 (a,b)
AUC_(0–t)_ (ng·h/mL)	28,813.4 ± 1406.97	4278.466 ± 127.72	41,848.64 ± 1115.51 (a)	8398.62 ± 457.89 (a)	82,233.89 ± 3608.25 (a,b)	19,046.64 ± 576.29 (a,b)
AUC_(0–∞)_ (ng·h/mL)	38,955.64 ± 2051.04	6328.87 ± 266.57	57,611.34 ± 1859.72 (a)	14,414.1 ± 1055.02	130,817.1 ± 7757.3 (a,b)	43,402.79 ± 6080.81 (a,b)
% Relative bioavailability (F_rel_)			145.240	196.299	285.401	445.175

Cmax: Maximum serum concentration, Tmax: time to reach cmax, AUC0-t: area under the serum concentration-time curve, MRT: mean residence time, ^a^ *p* < 0.05 relative to the oral MRZ suspension group, ^b^ *p* < 0.05 relative to the rectal free MRZ in situ gel group.

**Table 4:** Compartmental distribution of MRZ from rectal MRZ-NVMs in situ gel and oral MRZ suspension at different time intervals in male Wister rats.

Pharmacokinetics Parameter	Oral MRZ Suspension			Rectal MRZ-NVMs In Situ Gel Formulation		
Time (h)	Brain (ng/g)	Blood (ng/mL)	Br/Bl Ratio	Brain (ng/g)	Blood (ng/mL)	Br/Bl Ratio
0.5	166.868 ± 33.62	245.477 ± 12.19	0.6797	588.738 ± 55.22	624.87213 ± 19.72	0.9421
1	327.593 ± 37.24	481.047 ± 16.44	0.6809	1076.625 ± 96.92	1439.5543 ± 53.87	0.7479
2	568.346 ± 68.08	972.842 ± 34.06	0.5842	1636.367 ± 119.22	1961.9649 ± 65.92	0.834
4	502.618 ± 86.47	808.689 ± 29.59	0.6215	2043.617 ± 130.97	2616.9893 ± 156.38	0.7809
8	301.151 ± 16.50	700.806 ± 20.78	0.4297	1646.547 ± 134.33	1839.3004 ± 42.31	0.8952
12	210.556 ± 10.15	587.165 ± 23.06	0.3586	1332.3489 ± 108.72	1500.8905 ± 57.83	0.8877

## Data Availability

Data is contained within the article or Supplementary Material.

## Supplementary Materials

The following supporting information can be downloaded at: https://www.mdpi.com/article/10.3390/ph17080978/s1, Figure S1: % cumulative MRZ released from the 15 invasomal formulations compared to MRZ suspension; Figure S2: Cumulative MRZ permeated per unit area after 24h (Q24 µg/cm^2^) of all the invasomal formulations compared to MRZ suspension; Figure S3: Zeta potenial distibution curve for the optimized MRZ-NVM formulation; Figure S4: DSC theromgrams (A) and FT-IR spectrums (B) for MRZ and the mixtures of components of the invasomal formulation; Figure S5: Rheogram of the optimized MRZ-NVM in situ gel; Figure S6: Cumulative MRZ released % (A) and the cumulative amount of MRZ permeated after 24 h (B) for the optimized MRZ-NVM formulation and MRZ-NVM in situ gel; Figure S7: Results of toxicity study on the different hematologicals parameters and kidney and livir functions in different treatment groups after MRZ adminstration to animals; Figure S8: The histopathology of specific regions of the cerebral cortex in different treatment groups after MRZ administration to animals. (A) negative control group, (B) positive control group, (C) MRZ-susp (oral) group, (D) free MRZ gel (rectal) group, and (E) MRZ-NVM in situ gel (rectal); Figure S9: The histopathology of specific regions of the hippocampus in different treatment groups after MRZ administration to animals. (A) negative control group, (B) positive control group, (C) MRZ-susp (oral) group, (D) free MRZ gel (rectal) group, and (E) MRZ-NVM in situ gel (rectal); Figure S10: Effects of different treatments (Negative control, Positive control, MRZ-susp (oral), Free MRZ gel (rectal), and MRZ-NMV in situ gel (rectal) on the neurological activity and positive immune cells (%); Figure S11: HPLC separate chromotograms of (A) plain plasma, (B) Mirtazapine, and (C) Carbamazepine internal standard; Table S1: Regression analysis results for Y_1_, Y_2_, Y_3_ and Y_4_ responses; Table S2: Kinetic analysis of MRZ from different invasomal formulations together with MRZ suspension; Table S3: The predicted and experimental values of the four responses of the optimum MRZ-NVMs formulation.
