# Supplementary material for: pH-Sensitive In Situ Gel of Mirtazapine Invasomes for Rectal Drug Delivery: Protruded Bioavailability and Anti-Depressant Efficacy
Source: Pharmaceuticals (Basel). 2024 Jul 24;17(8):978. doi: 10.3390/ph17080978 (PMC11357403; doi:10.3390/ph17080978)

Supplementary Figure S1

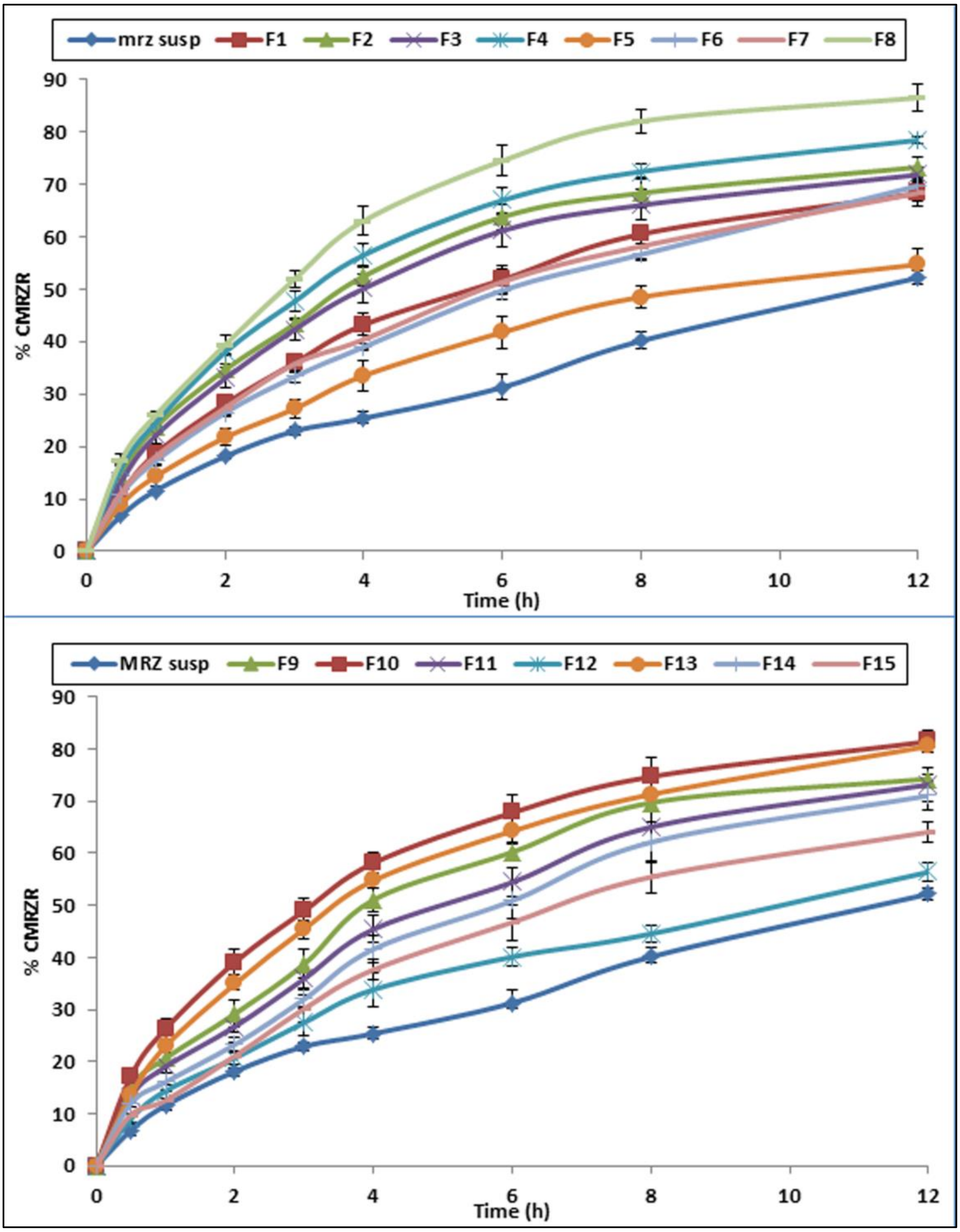

Supplementary Figure S2

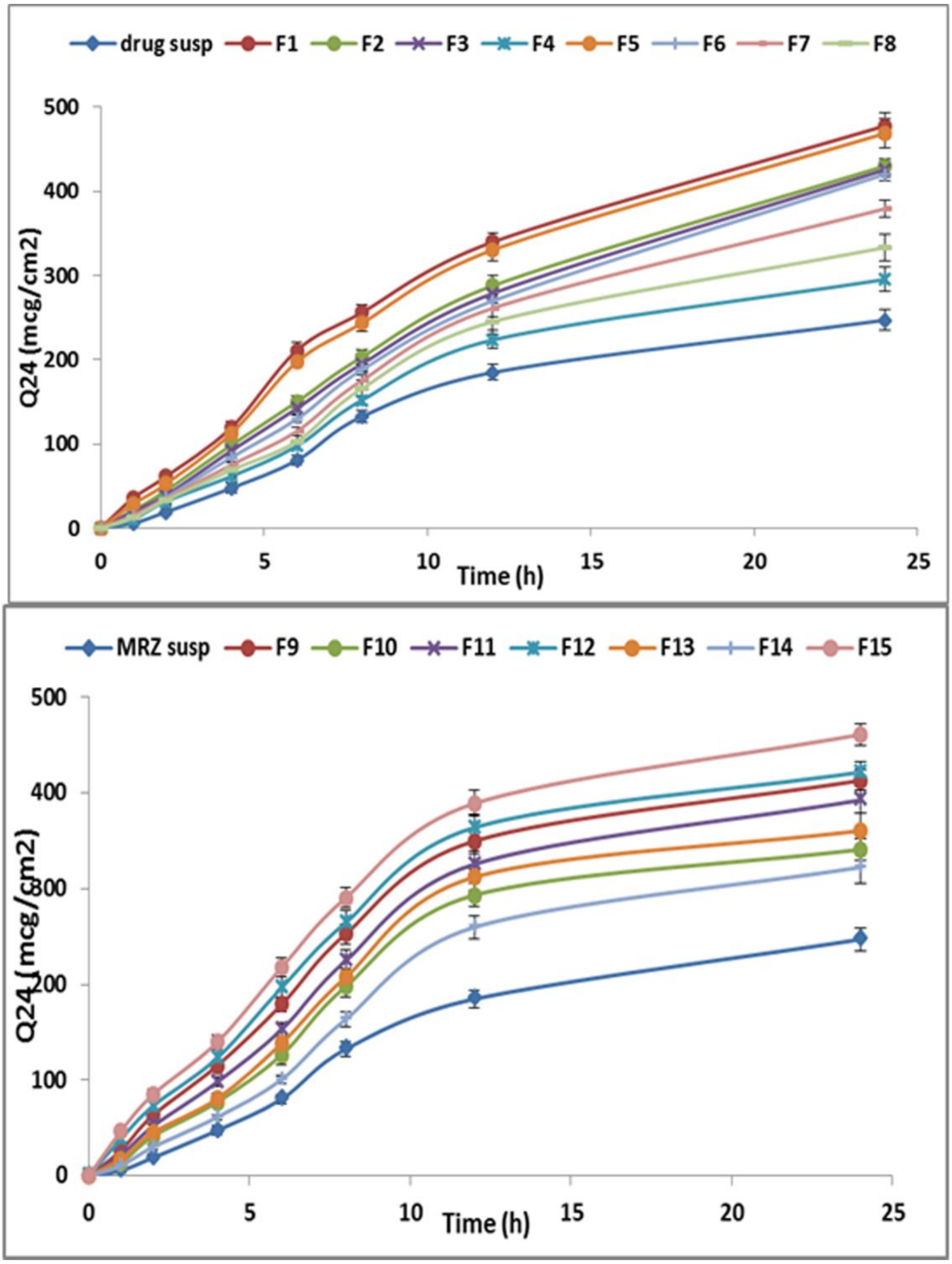

Supplementary Figure S3

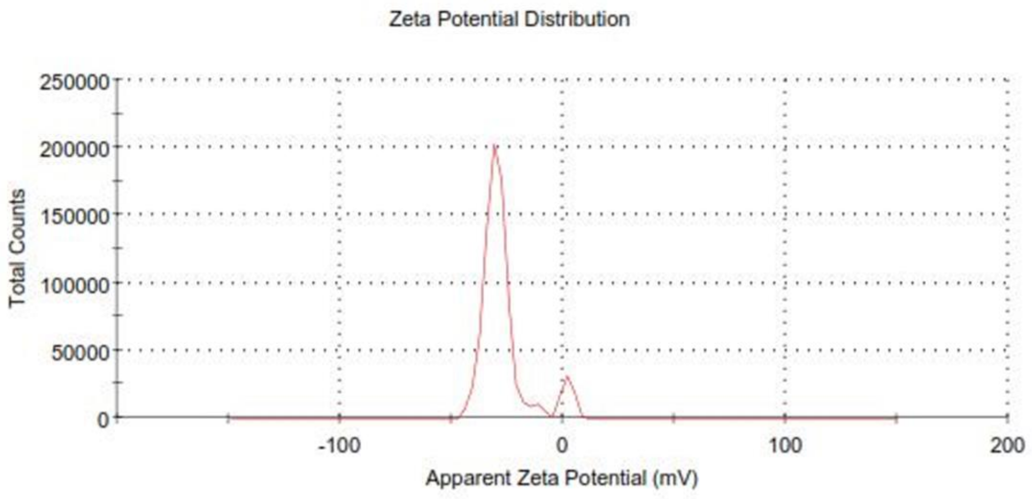

Supplementary Figure S4

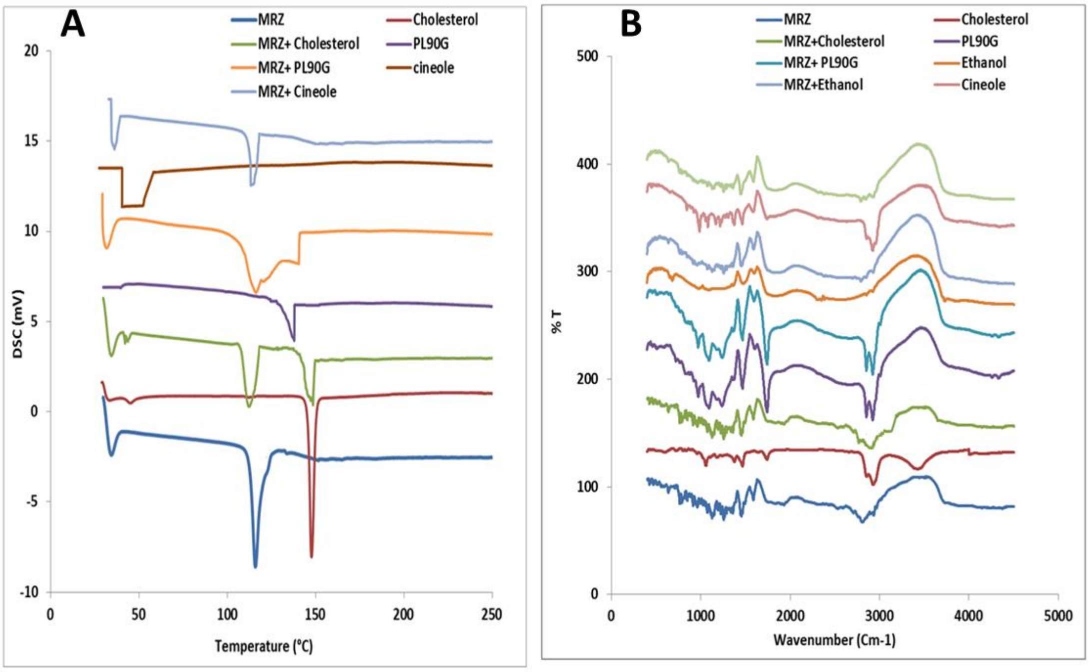

Supplementary Figure S5

pH	viscosity		Farrow's constant N	Flow behavior	Area of hysteresis loop ($dyne/cm^2.sec$)
	Min (cP)	Max (cP)			
**4**	370.5	1280.03	2.61	Shear rate thinning (pseudoplastic)	1889.776
**7.4**	1206.88	7218.08	2.83	Shear rate thinning (pseudoplastic)	2696.528

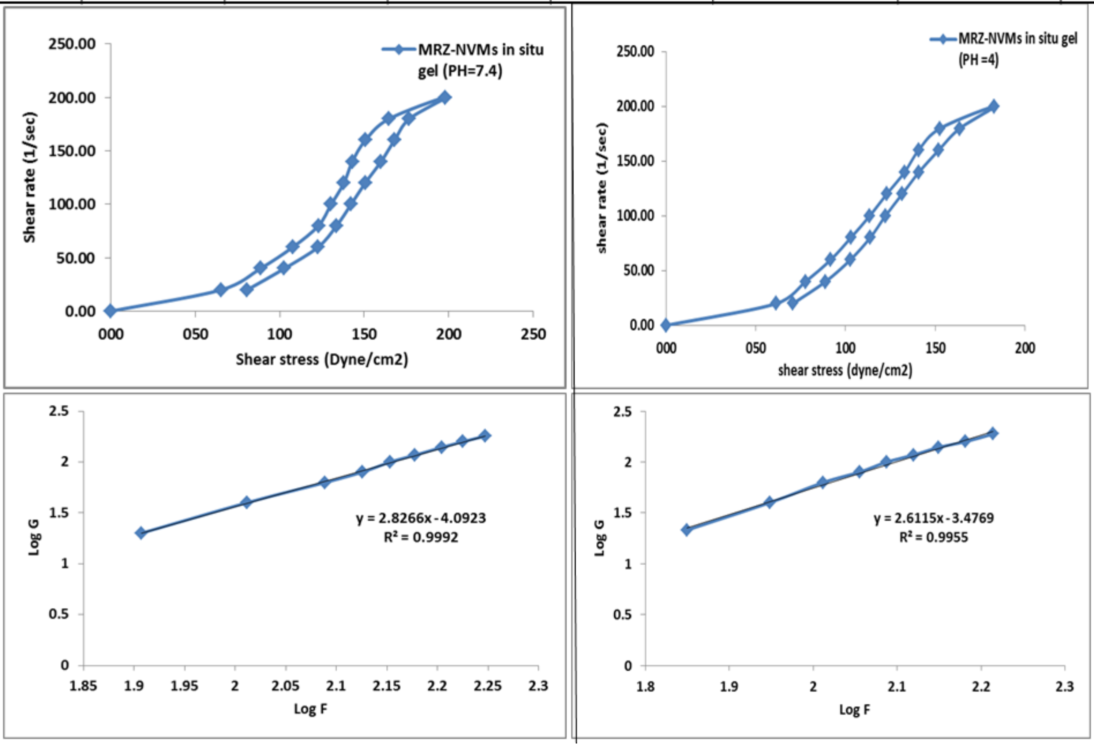

Supplementary Figure S6

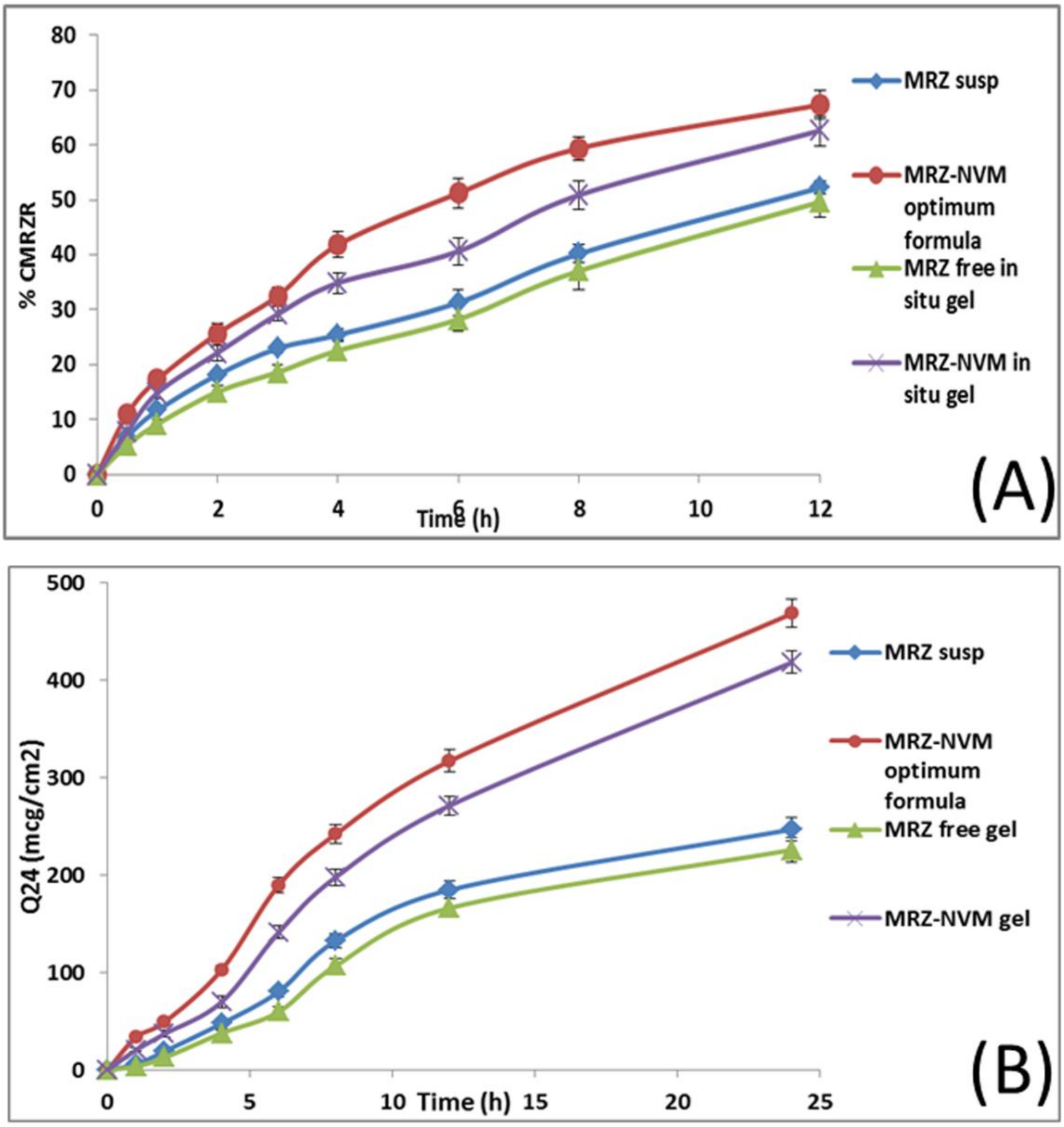

Supplementary Figure S7

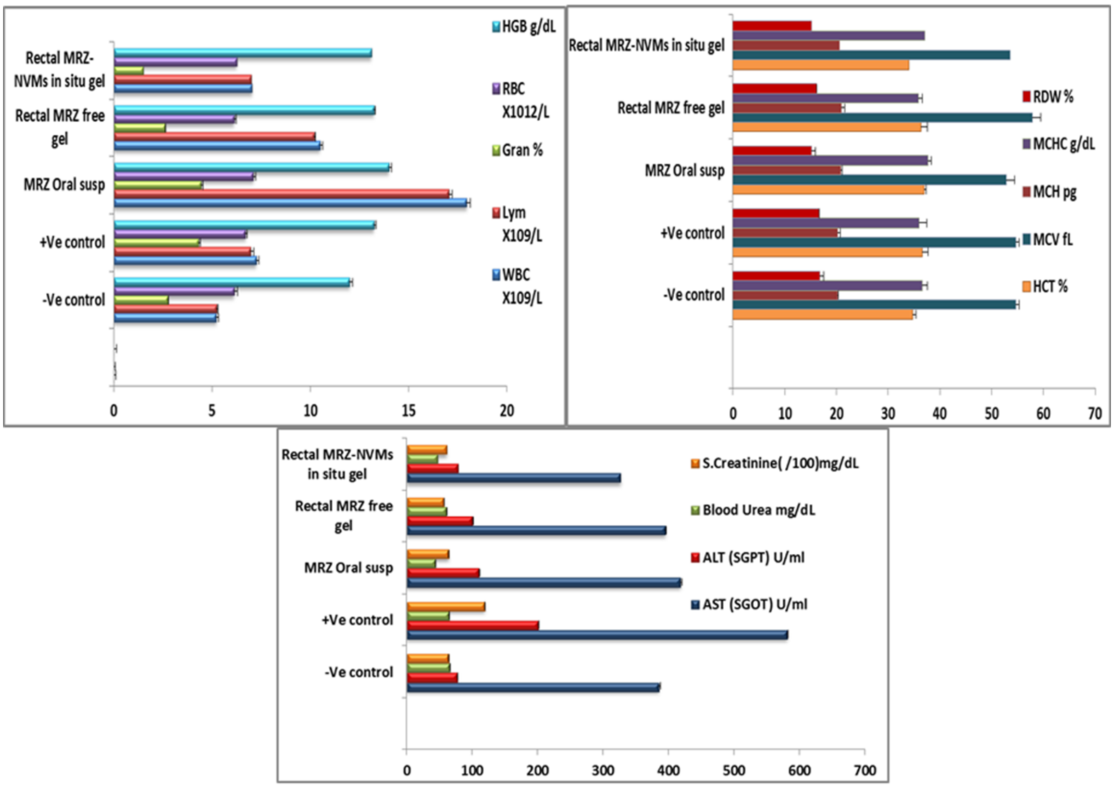

Supplementary Figure S8

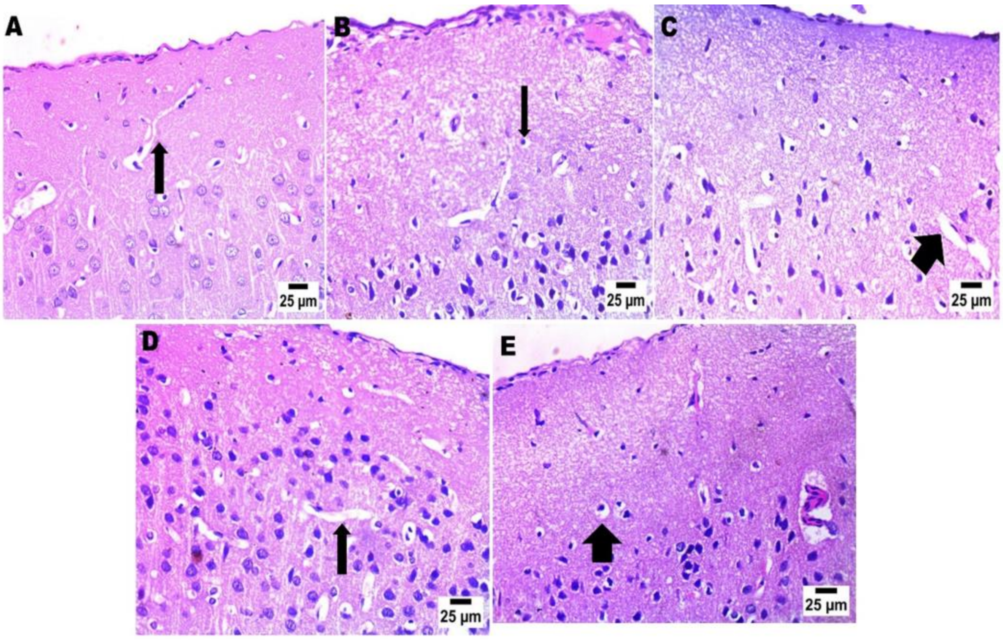

Supplementary Figure S9

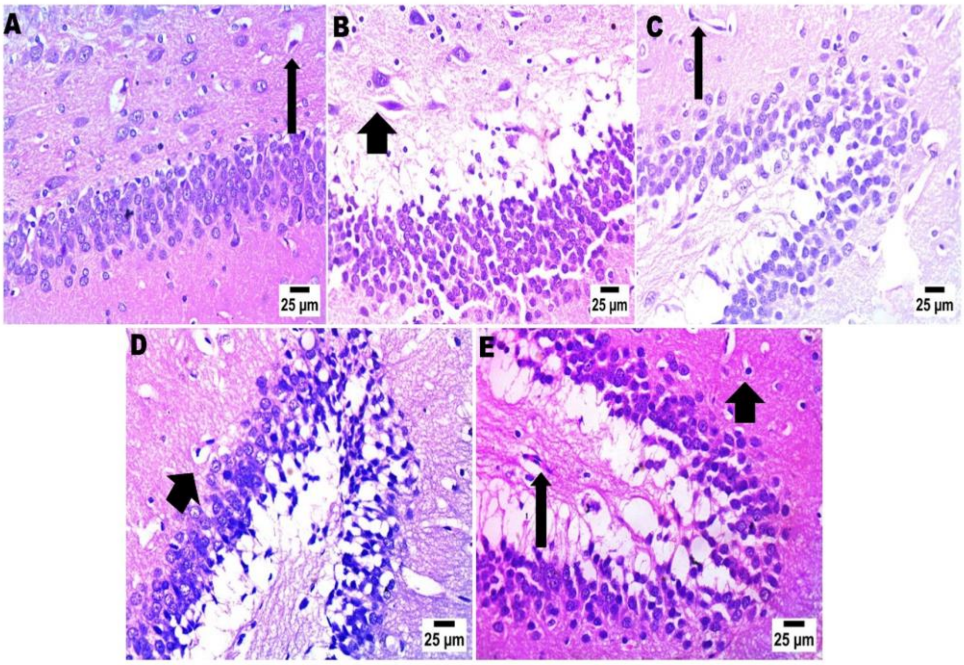

Supplementary Figure S10

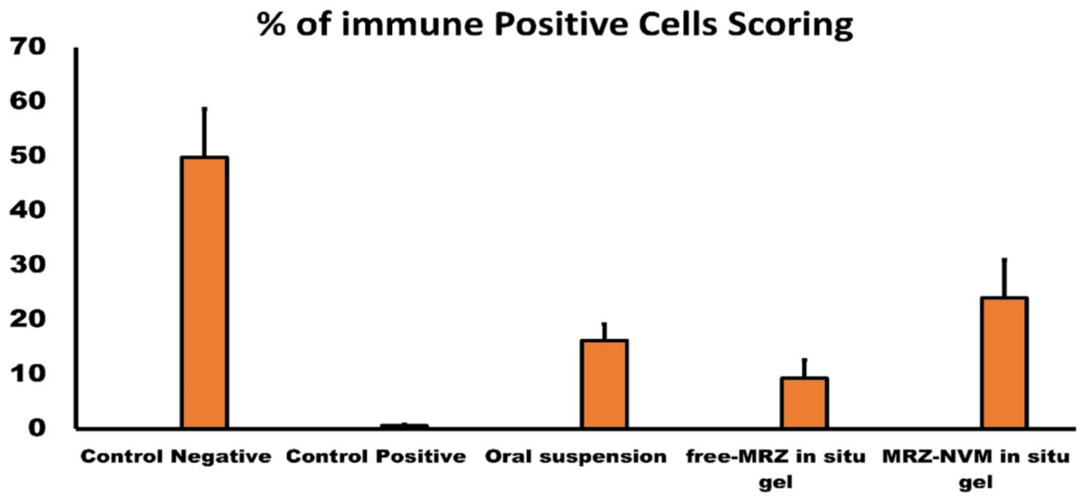

Supplementary Figure S11

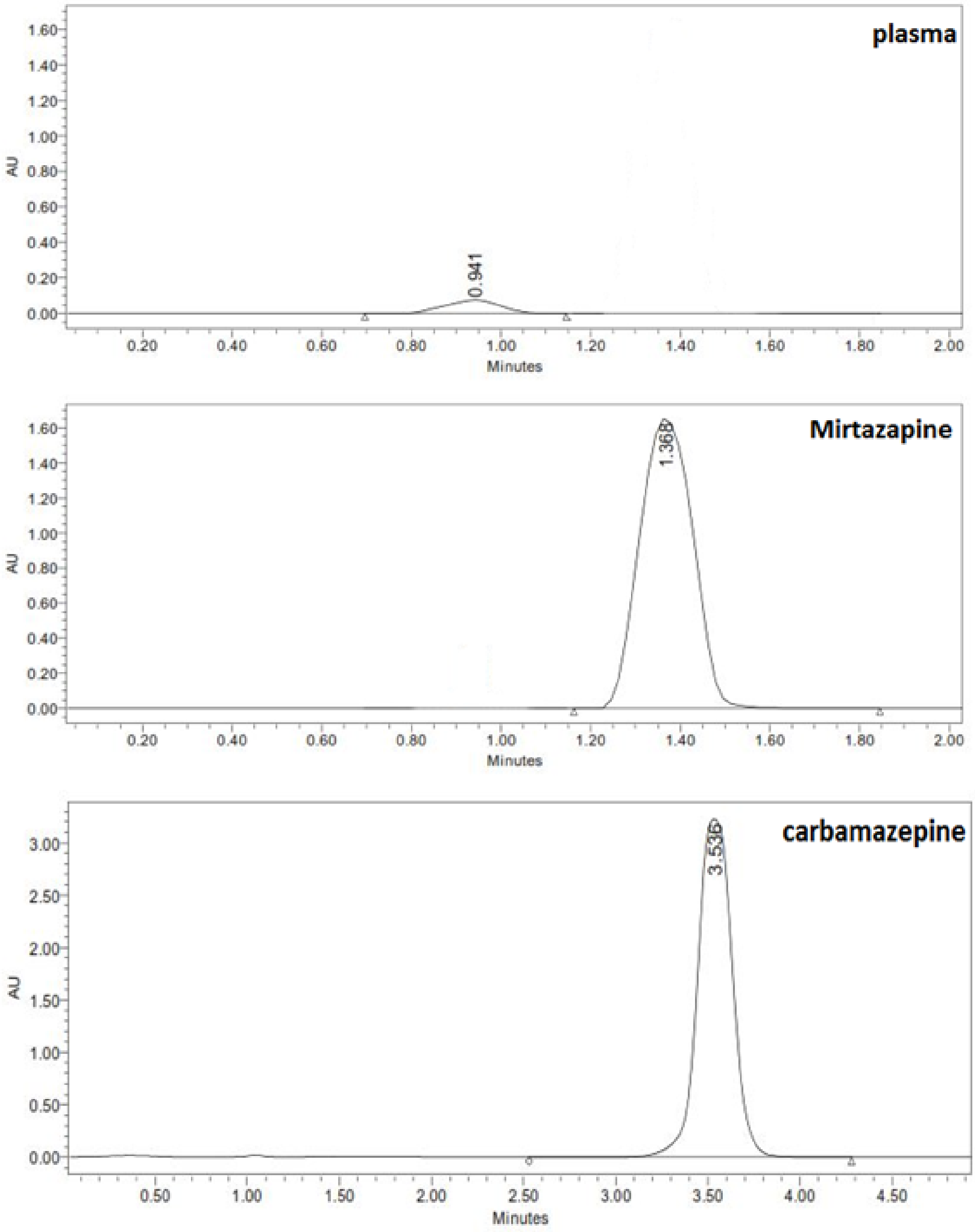

Supplement: Supplementary file 1 [file pharmaceuticals-17-00978-s001.zip › pharmaceuticals-3087194-supplementary/Figures S1-S11.pdf]
