# Supplementary material for: pH-Sensitive In Situ Gel of Mirtazapine Invasomes for Rectal Drug Delivery: Protruded Bioavailability and Anti-Depressant Efficacy
Source: Pharmaceuticals (Basel). 2024 Jul 24;17(8):978. doi: 10.3390/ph17080978 (PMC11357403; doi:10.3390/ph17080978)
Supplement: Supplementary file 1 [file pharmaceuticals-17-00978-s001.zip › pharmaceuticals-3087194-supplementary/Tables S1-S3.pdf]

Supplementary Table S1: Regression analysis results for Y_1, Y_2, Y_3 and Y_4 responses

Quadratic model	Adequate precision	R^2	Adjusted R^2	Predicted R^2	SD	% CV	P value
**Response Y_1**	33.303	0.9661	0.9571	0.9388	2.44	3.14	<0.0001
**Response Y_2**	66.239	0.9908	0.9884	0.9843	6.54	3.12	<0.0001
**Response Y_3**	29.866	0.9570	0.9457	0.9226	416.38	5.93	<0.0001
**Response Y_4**	32.050	0.9669	0.9581	0.9399	1.094E+006	7.42	<0.0001

*R^2: coefficient of determination; SD: standard deviation; CV: coefficient of variation.

Supplementary Table S2: Kinetic analysis of MRZ release from different MRZ-NVMs formulations together with MRZ suspension.

Formulation	R2			Mechanism
	Zero	First	Higuchi	
**F1**	0.8976	0.9688	**0.9981**	Diffusion
**F2**	0.8349	0.9261	**0.9805**	Diffusion
**F3**	0.849	0.9359	**0.9868**	Diffusion
**F4**	0.8359	0.9435	**0.9961**	Diffusion
**F5**	0.9173	0.9672	**0.9949**	Diffusion
**F6**	0.9166	0.9789	**0.9989**	Diffusion
**F7**	0.9179	0.9831	**0.9966**	Diffusion
**F8**	0.8385	0.9587	**0.9905**	Diffusion
**F9**	0.8724	0.9513	**0.9864**	Diffusion
**F10**	0.8424	0.9598	**0.9989**	Diffusion
**F11**	0.9112	0.9799	**0.9941**	Diffusion
**F12**	0.9246	0.9724	**0.9925**	Diffusion
**F13**	0.8627	0.9645	**0.9993**	Diffusion
**F14**	0.9308	0.9861	**0.9919**	Diffusion
**F15**	0.94	0.9805	**0.9958**	Diffusion
**MRZ susp**	0.97014	**0.9897**	0.9797	First
**MRZ-NVM optimum formula**	0.91175	0.97613	**0.99921**	Diffusion
**MRZ-free gel**	0.97991	**0.99389**	0.98587	First
**MRZ-NVM gel**	0.944886	0.988271	**0.990142**	Diffusion

Supplementary Table S3: The predicted and experimental values of the four responses of theoptimum MRZ-NVMs formulation.

Solution	PL90G (%w/v)	Cineole (%v/v)	Ethanol (%v/v)	Y_1 (%)	Y_2 (nm)	Y_3 (%)	Y_4 (µg/cm²)	desirability
**Predicted Value**	4.81	0.5	5	83.70	221.87	68.79	471.48	0.553
**Experimental Value**	4.81	0.5	5	81.45 ± 0.53	201.3 ± 4.71	67.29 ± 2.54	468.68 ± 14.31	0.553
**Bias%**	-	-	-	2.76	10.22	2.24	0.559	-

Each single value represents the average ± (SD) (n=3).
%Bias = (predicted value-Experimental value)/Experimental value
